# Histone lysine methacrylation is a dynamic post-translational modification regulated by HAT1 and SIRT2

**DOI:** 10.1038/s41421-021-00344-4

**Published:** 2021-12-28

**Authors:** Kyle Delaney, Minjia Tan, Zhesi Zhu, Jinjun Gao, Lunzhi Dai, Sunjoo Kim, Jun Ding, Maomao He, Levon Halabelian, Lu Yang, Prabakaran Nagarajan, Mark Robert Parthun, Sangkyu Lee, Saadi Khochbin, Yujun George Zheng, Yingming Zhao

**Affiliations:** 1 Ben May Department for Cancer Research, The University of Chicago, Chicago, IL USA; 2 Department of Pharmaceutical and Biomedical Sciences, University of Georgia, Athens, GA USA; 3 College of Pharmacy, Research Institute of Pharmaceutical Sciences, Kyungpook National University, Daegu, South Korea; 4 Structural Genomics Consortium, University of Toronto, Toronto, ON Canada; 5 Department of Biological Chemistry and Pharmacology, The Ohio State University, Columbus, OH USA; 6 CNRS UMR 5309, INSERM, U1209, Université Grenoble Alpes, Institute for Advanced Biosciences, Grenoble, France; 7 Present Address: Shanghai Institute of Materia Medica, Chinese Academy of Sciences, Shanghai, China; 8 Present Address: Department of General Practice, State Key Laboratory of Biotherapy, West China Hospital, Sichuan University, and Collaborative Innovation Center of Biotherapy, Chengdu, China

**Keywords:** Post-translational modifications, Proteomics

## Abstract

Histone lysine crotonylation is a posttranslational modification with demonstrated functions in transcriptional regulation. Here we report the discovery of a new type of histone posttranslational modification, lysine methacrylation (Kmea), corresponding to a structural isomer of crotonyllysine. We validate the identity of this modification using diverse chemical approaches and further confirm the occurrence of this type of histone mark by pan specific and site-specific anti-methacryllysine antibodies. In total, we identify 27 Kmea modified histone sites in HeLa cells using affinity enrichment with a pan Kmea antibody and mass spectrometry. Subsequent biochemical studies show that histone Kmea is a dynamic mark, which is controlled by HAT1 as a methacryltransferase and SIRT2 as a de-methacrylase. Altogether, these investigations uncover a new type of enzyme-catalyzed histone modification and suggest that methacrylyl-CoA generating metabolism is part of a growing number of epigenome-associated metabolic pathways.

## Introduction

The covalent binding of chemical groups from reactive intermediate metabolites to form protein posttranslational modifications (PTMs) has emerged as an important mechanism for regulating cellular processes^1,2^. Histone proteins carry a diverse range of PTMs, which are also known as histone marks, originating from a variety of metabolic pathways^3,4^. The formation of PTMs can occur through either spontaneous chemical reactions or enzymatic reactions^5,6^. Highly energetic metabolites, such as acyl CoAs or S-Adenosyl methionine (SAM), are typical cofactors for enzyme-catalyzed PTM reactions^7^.

One of the most studied types of histone marks is lysine acetylation, which is derived from the metabolite acetyl-CoA. However, acetyl-CoA is not the only acyl-CoA capable of forming histone marks. An increasing number of new types of histone marks derived from a variety of acyl-CoA species have been discovered in recent years^4,8,9^. One such example is lysine crotonylation, an enzymatically regulated epigenetic modification associated with active gene expression^10,11^. Since the initial report of its discovery, both crotonyltransferases and de-crotonylases have been identified^10–16^. It has also been shown that some protein modules recognize and bind with better affinity to histone crotonyllysine marks than their corresponding histone acetyllysine marks^17–20^. Diverse studies indicate that histone lysine crotonylation is involved in the transcriptional regulation^10,11,14,15,21,22^. Although multitudes of acyl-CoA metabolite species are involved in cell metabolism, to what extent these other acyl-CoA metabolites are used in the generation of enzymatically regulated histone modifications remains poorly understood.

Mounting evidence suggests that previously classified acetyltransferases and deacetylases also regulate non-acetyl acyl modifications. Indeed, some enzymes appear to be more active for the recently identified lysine acylations. For instance, we previously found that SIRT5 specifically targets the negatively charged lysine acylations^23,24^, a finding which is consistent with the work of others^25^. In other cases, enzymes that regulate lysine acetylation also have promiscuous activity for non-acetyl acylations. For example, p300 is an acyltransferase that is capable of utilizing a variety of acyl-CoA substrates^26^. Notably, a proteomics study shows that p300 has unique sets of protein substrates for protein lysine acetylation and lysine 2-hydroxyisobutyrlation, suggesting this acyltransferase exhibits acylation-dependent substrate selectivity^27^. Furthermore, p300 regulates glycolysis through lysine 2-hydroxyisobutyrlation of glycolytic enzymes rather than acetylation of these enzymes^27^. As such, understanding the role of previously classified acetyltransferases and deacetylases in regulating lysine acylations will help us to have a more thorough understanding of their roles in cellular regulations.

Here we report the identification of a new type of histone posttranslational modification, methacryllysine (Kmea). Kmea is a structural isomer of crotonyllysine (Kcr). We validated the identity of Kmea as being separate from Kcr using HPLC and MS/MS analysis of synthetic peptides and ozonolysis reactions. Furthermore, we confirmed that treatment of cells with methacrylate led to direct incorporation of the metabolite into this modification using isotopic labeling. In total, we identified 27 Kmea modified histone sites in HeLa cells using affinity enrichment with a pan Kmea antibody and mass spectrometry. We also found that HAT1 catalyzes the addition of the Kmea on histone H4 peptide in vitro and ex vivo. We also found that the class III deacetylases SIRT1 and SIRT2 catalyze the removal of Kmea. In summary, we provide the first evidence of Kmea as a new type of posttranslational modification and identify enzymes involved in its addition and removal. In this context, we also demonstrate the ability of HAT1 to catalyze histone lysine methacrylation, reclassifying this enzyme among the growing family of acyltransferase.

## Results

### Discovery and validation of methacryllysine (Kmea)

**Fig. 1:**
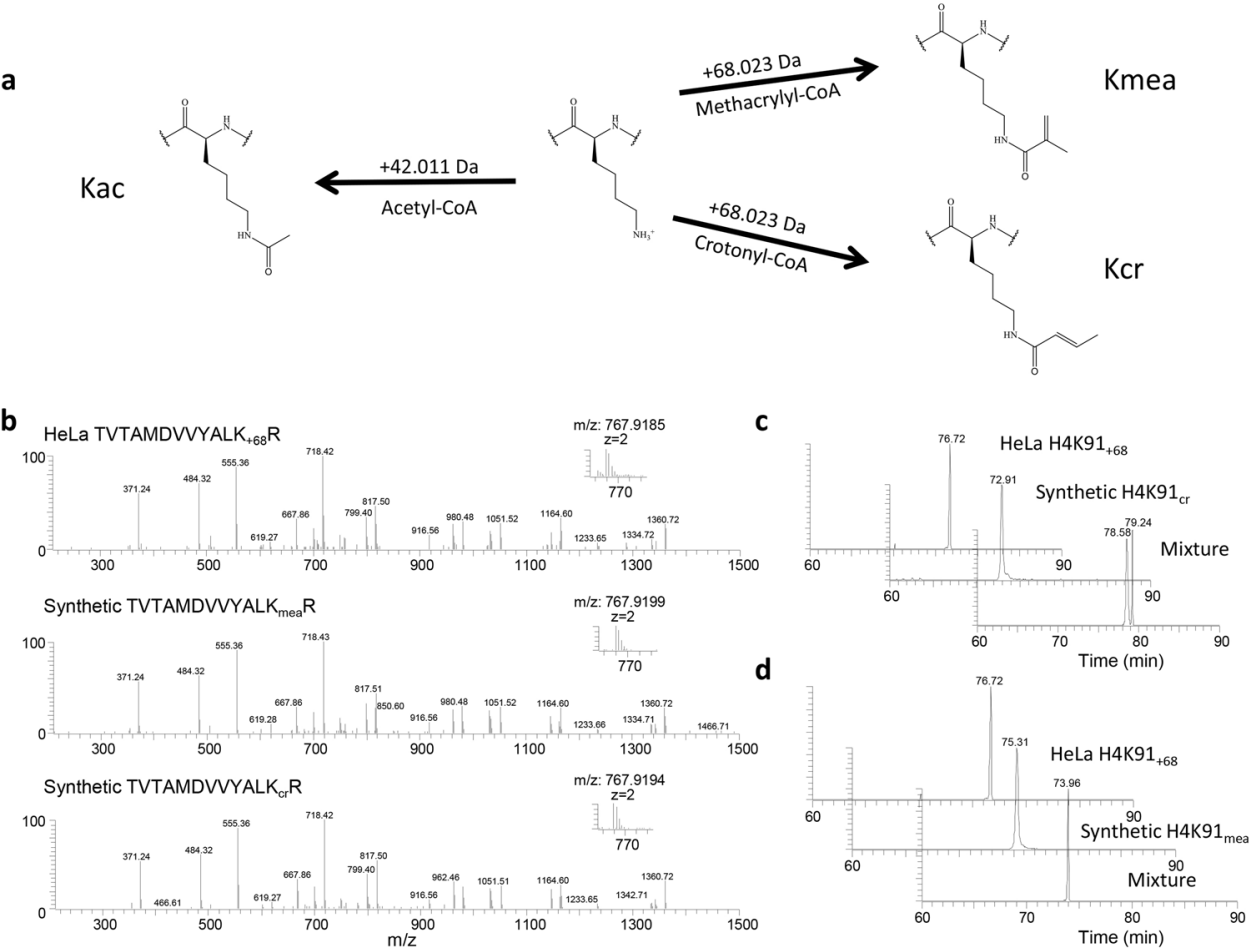
Identification and verification of lysine methacrylation (Kmea). **a** Lysine (center) is capable of being modified with multiple different PTMs depending on which acyl-CoA is used in the enzymatic reaction. The appropriate acyl-CoAs as well as the associated mass shift added to a lysine residue is indicated for acetyllysine (left), methacryllysine (Kmea, upper right), and crotonyllysine (Kcr, lower right). **b** MS/MS spectra of an exemplary HeLa histone peptide (top), synthetic H4K91ma peptide (middle), and synthetic H4K91cr peptide (bottom). **c** Extraction ion chromatograms of HeLa cells-derived histone peptide (TVTAMDVVYALK_+68_R) (top), synthetic H4K91cr peptide (middle), and a mixture of the two peptides (bottom). **d** Extraction ion chromatograms of HeLa cells-derived histone peptide (TVTAMDVVYALK_+68_R) (top), synthetic H4K91mea peptide (middle), and a mixture of the both peptides (bottom).

Based on the ability of various acyl-CoA species to mediate the corresponding lysine acylations, we hypothesized that the endogenous metabolite methacrylyl-CoA would also mediate the formation of methacryllysine (Kmea) (Fig. 1a). Kmea is a structural isomer of crotonyllysine (Kcr), a modification that we previously discovered^11^. To narrow our search for potential Kmea sites, we focused on those histone lysine sites with a mass shift of +68.023 Da in HeLa cells that were not labeled by isotopic crotonate in our previous work^11^. This provided us with multiple candidates for which we generated synthetic peptides.

We extracted histones from HeLa cells by acid extraction method and then subjected the samples to tryptic digestion. We analyzed these samples using HPLC/MS/MS. We compared the fragmentation patterns for histones with +68.023 Da mass shifts on histone H4 lysine 91 (H4K91) from HeLa histones to synthetic peptides with either the Kcr or Kmea modifications (Fig. 1b). As an example, the MS/MS pattern is shown for an H4 peptide identified by HPLC/MS/MS analysis of tryptic peptides derived from HeLa core histones with the peptide sequence TVTAMDVVYALK_+68_R (Fig. 1b, top). This ex vivo derived peptide has a nearly identical MS/MS pattern as its corresponding synthetic Kcr and Kmea peptides (Fig. 1b, middle and bottom).

To confirm the identity of the peptide from HeLa histones, we performed co-elution experiments. In co-elution, it is known that any peptides that can be separated by chromatography are confirmed to have distinct chemical structures. We found that the synthetic H4K91cr peptide did not co-elute with the H4K91 + 68.023 peptide derived from HeLa cells (Fig. 1c), confirming that the cell-derived peptide was not modified with Kcr. In contrast, we found that the H4K91 + 68.023 Da peptide from HeLa cells co-eluted with the synthetic H4K91mea peptide (Fig. 1d), supporting its identification as a Kmea peptide. We similarly validated H4K31mea using the same approach (Supplementary Fig. S1).

**Fig. 2:**
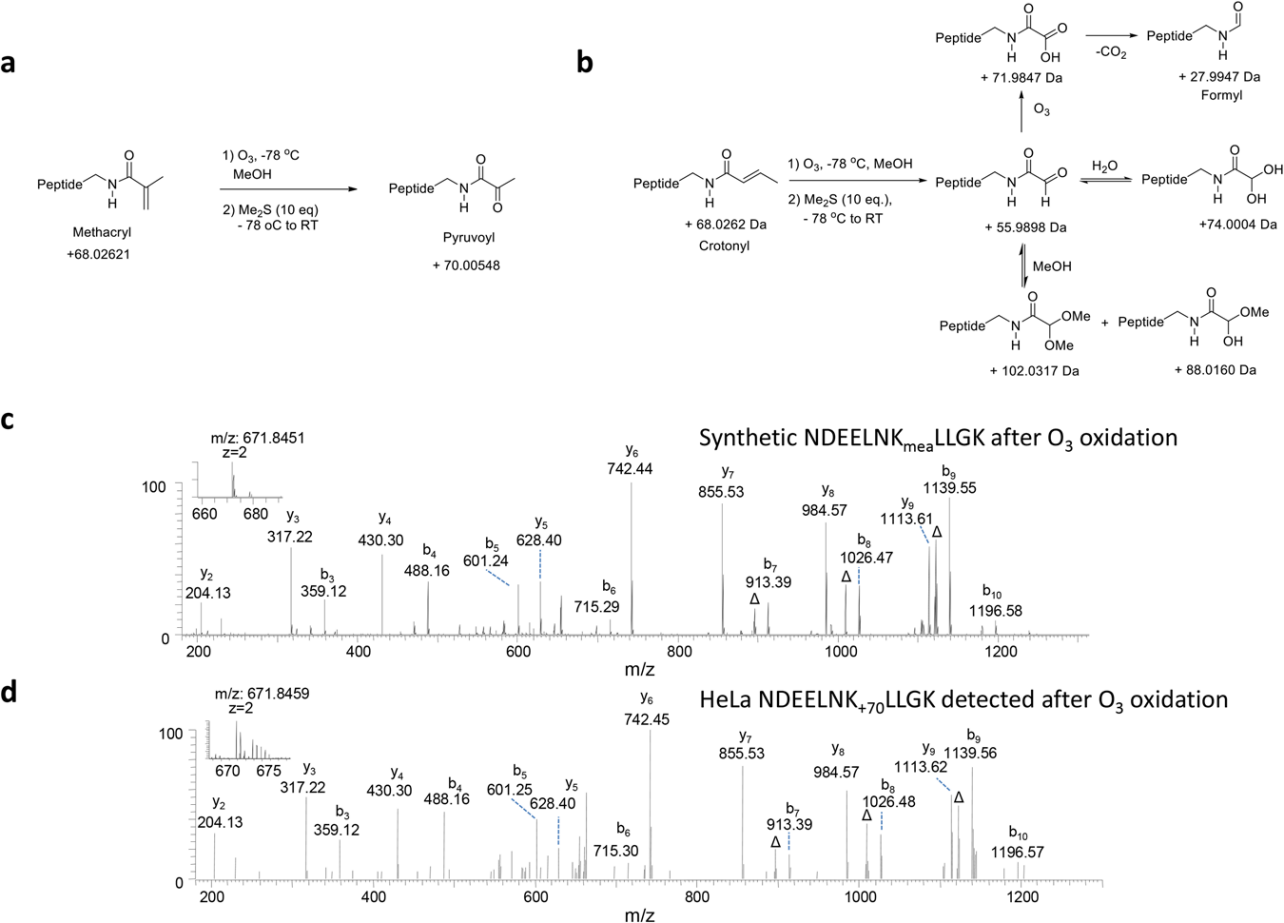
Verification of Kmea structure by ozone (O3) oxidation. **a**, **b** Chemical reactions for ozonolysis of the double bonds in Kmea (**a**) and Kcr (**b**) peptides. **c** MS/MS of ozonolyzed peptides. The synthetic H2AK95mea peptide, NDEELNKmeaLLGK, was subjected to ozonolysis reaction. Its main reaction product, NDEELNK_+70_LLGK, was analyzed by HPLC/MS/MS analysis. d MS/MS of a NDEELNK _+70_LLGK peptide detected in O3 oxidized HeLa histone extract.

The co-elution experiments could not unambiguously validate two peptides, an H2A peptide (NDEELNK_+68_LLGK) and an H4 peptide (GVLK_+68_VFLENVIR) as either Kcr or Kmea modification (Supplementary Fig. S2), because the ex vivo-derived peptides co-eluted with their corresponding Kcr and Kmea peptides. To differentiate the two modifications, we took advantage of the unique double bond in either Kcr or Kmea, and ozonolysis chemistry. In an ozonolysis reaction, ozone cleaves molecules at unsaturated bonds. Structural differences between Kmea and Kcr modifications lead to products with different masses resulting from the reaction (Fig. 2a, b). Ozonolysis of a Kcr-modified peptide generates its corresponding pyruvoyllysine-modified peptide that has a mass shift of +70.005 Da (Fig. 2a). On the other hand, ozonolysis of a crotonyllysine-modified peptide oxidizes and cleaves the double bond in the crotonyl group, leading to the production of multiple possible products (Fig. 2b).

To differentiate the two possibilities, we carried out ozonolysis reaction for the ex vivo-derived peptides with a mass shift of +68 Da and their synthetic counterparts bearing either Kcr or Kmea modification. We measured the reaction products by HPLC/MS/MS to validate the identity of the posttranslational modification isolated from cells. As expected, ozonolysis of the synthetic H2AK95mea peptide, NDEELNK_mea_LLGK, resulted in a pyruvoylated product with a mass shift of +70 Da. Importantly, MS/MS spectra of ozonolytic products from synthetic NDEELNK_mea_LLGK, and its corresponding ex vivo-derived peptide, both of which have a mass shift of +70 Da, are nearly identical (Fig. 2c, d). Likewise, we carried out the similar experiment for synthetic H2AK95cr peptide, NDEELNK_cr_LLGK. Ozonolysis of the peptides produced multiple products, including two major oxidized peptides with mass shifts of +28 and +72 Da (Supplementary Fig. S3), which correspond to the masses of partially and complete oxidized products of the Kcr-modified peptide (Fig. 2a). In summation, the ex vivo derived H2AK95 + 68 Da peptide generated the same ozonolytic product as its corresponding Kmea peptide, but not Kcr peptide. Thus, the mass shift in the HeLa H2AK95 + 68.023 Da modified peptide is caused by Kmea instead of Kcr. Likewise, we validated the identity of H4K59mea by this method (Supplementary Fig. S4). In summary, a comparison between four ex vivo*-*derived histone peptides with their corresponding synthetic peptides, with or without ozonolysis reaction, identified Kmea as a new type of posttranslational modification on histones.

### Validation of Kmea by immunochemistry

**Fig. 3:**
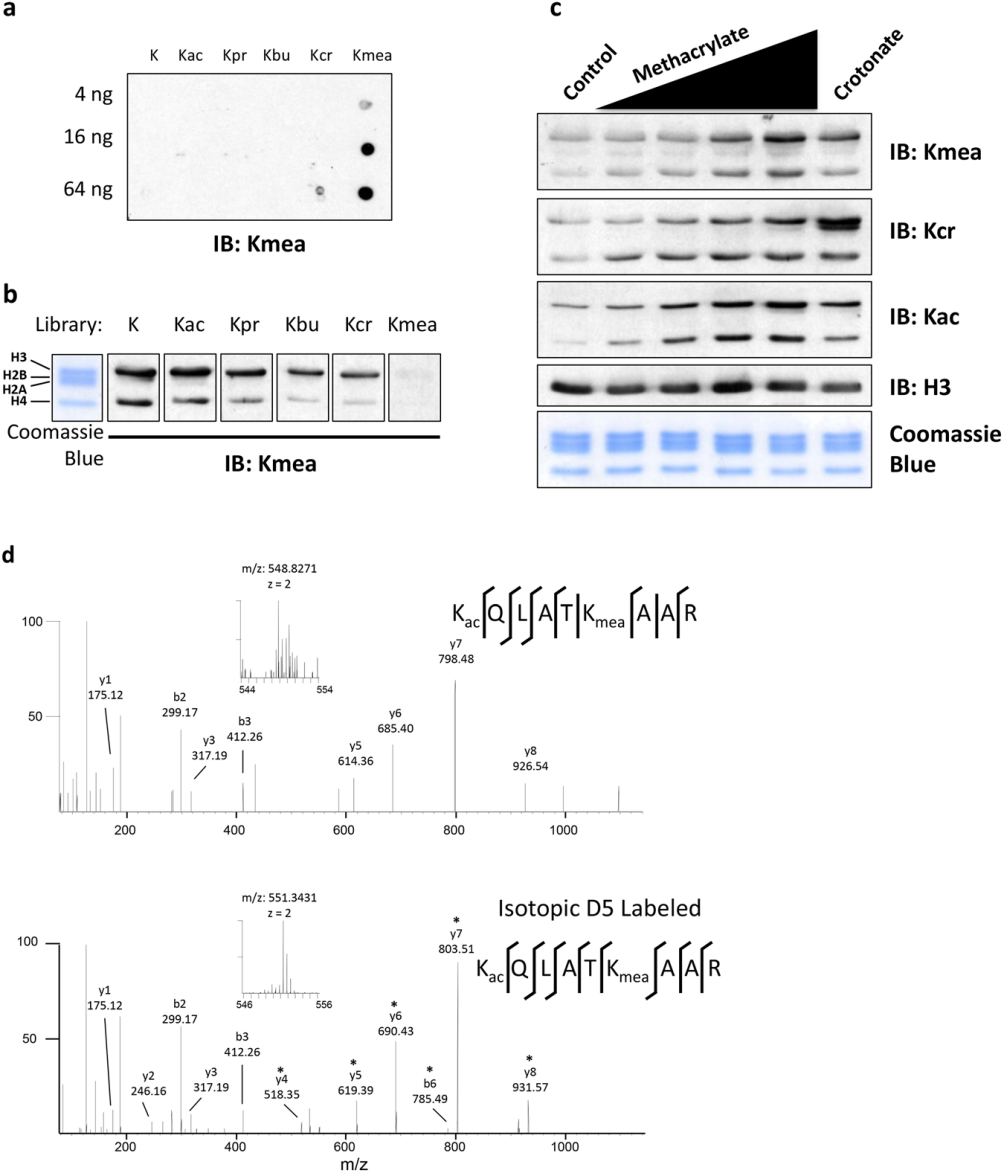
Methacrylate is a metabolic precursor for Kmea. **a** Dot blot assay used to assess specificity of a pan anti-Kmea antibody. The Kmea peptide library consists of peptides with the sequence CXXXXXKmeaXXXXX where C is cysteine, X is a mixture of the 19 non-cysteine amino acids, and Kmea is methacryllysine. The control peptides libraries differ in modification status of the central lysine residue where the modifications are abbreviated as follows: K, unmodified lysine; Kac, acetyllysine; Kpr, propionyllysine; Kbu, butyryllysine; and Kcr, crotonyllysine. The amount of peptides spotted in each row of the membrane is indicated. **b** Western blotting was performed using a pan anti-Kmea antibody on acid extracted HeLa histones. The pan anti-Kmea antibody was pre-incubated with peptide libraries for 4 h prior to immunoblotting of the western blot membrane. The sequences and abbreviations of the peptide libraries are the same as those indicated for the dot blot in **a**. **c** Western blot results for histones extracted from HeLa cells. HeLa cells were treated for 24 h with 1, 3, 5, or 10 mM sodium methacrylate or 10 mM sodium crotonate. **d** MS/MS spectra of H3K23mea peptide from HeLa histones following treatment with either methacrylate (top) or d-7 methacrylate (bottom). Asterisks (*) indicate b and y ions with 5 Da mass shift consistent with incorporation of the isotopic metabolite.

To further validate histone Kmea, we generated a polyclonal pan anti-Kmea antibody. By dot blot analysis, we found that the Kmea antibody had 20-fold or more specific binding activity for a Kmea peptide library than the other acylated peptide libraries we tested (Fig. 3a). Furthermore, this pan anti-Kmea antibody detected Kmea on the acid extracted histones from HeLa cells (Fig. 3b). We performed a competition assay by incubating the pan anti-Kmea antibody in the presence of various peptide libraries. The immunoblot signal was competed away using a synthetic Kmea peptide library to a greater extent than when competition was performed with any other tested synthetic peptide library with unmodified, acetylated (Kac), propionylated (Kpr), butyrylated (Kbu), or crotonylated (Kcr) lysines (Fig. 3b). This experiment confirms that the signal we detected by western blot was due to the presence of antibodies in the polyclonal mixture that are specific for the Kmea peptide library. We similarly generated site- specific anti-H4K5mea and anti-H3K18mea antibodies and validated their specificity by dot blot (Supplementary Fig. S5a, b).

### Methacrylate is a metabolic precursor for histone Kmea

Acetate and crotonate can be used by cellular short-chain CoA synthetases for the generation of their corresponding CoAs, the cofactors for lysine acylation reactions. We hypothesized that methacrylyl-CoA would serve as the cofactor for lysine methacrylation. Similar to the effects of acetate and crotonate, we hypothesized that treatment of HeLa cells with sodium methacrylate would induce the generation of methacrylyl-CoA that is in turn used by acyltransferases for the formation of Kmea. Consistent with our hypothesis, we found that increasing doses of sodium methacrylate coincided with increases in histone Kmea signal (Fig. 3c). Furthermore, the effect of equimolar doses of methacrylate and crotonate had the strongest effects on increasing signal on Kmea and Kcr, respectively (Fig. 3c). In response to methacrylate treatment, we found similar effects on H4K5mea and H3K18mea visualized by western blot using the corresponding specific antibodies (Supplementary Fig. S5c).

It is of note that the treatment of cells by sodium methacrylate also induced H3 and H4 acetylation and crotonylation to some extent (Fig. 3c and Supplementary Fig. S5c). Since the tested antibodies were highly specific by dot blot analysis, these data suggest that methacrylate may somehow affect the production or availability of acetyl and crotonyl-CoA or that it may interfere with the activity of HDACs that remove the acetyl and crotonyl marks. It is known that certain short chain fatty acids, most notably butyrate, lead to increases in Kac by acting as histone deacetylase inhibitors^28^. Additionally, crotonate has also been reported to inhibit HDACs in vitro^21^.

To confirm that methacrylate was capable of serving as a metabolic precursor for Kmea. We treated HeLa cells with d7-labeled methacrylate for 24 h before isolating histones through acid extraction. We then tryptically digested the histones and enriched Kmea sites using the pan anti-Kmea antibody. Using this approach, we detected three sites where the isotopic methacrylate was incorporated into the Kmea modification (Fig. 3d, Supplementary Figs. S6, S7).

These results clearly demonstrate that methacrylate can serve as a metabolic precursor for histone Kmea. Furthermore, these results further validate the existence of Kmea as a unique modification that occurs independently of Kcr.

### Mapping histone Kmea sites

**Fig. 4:**
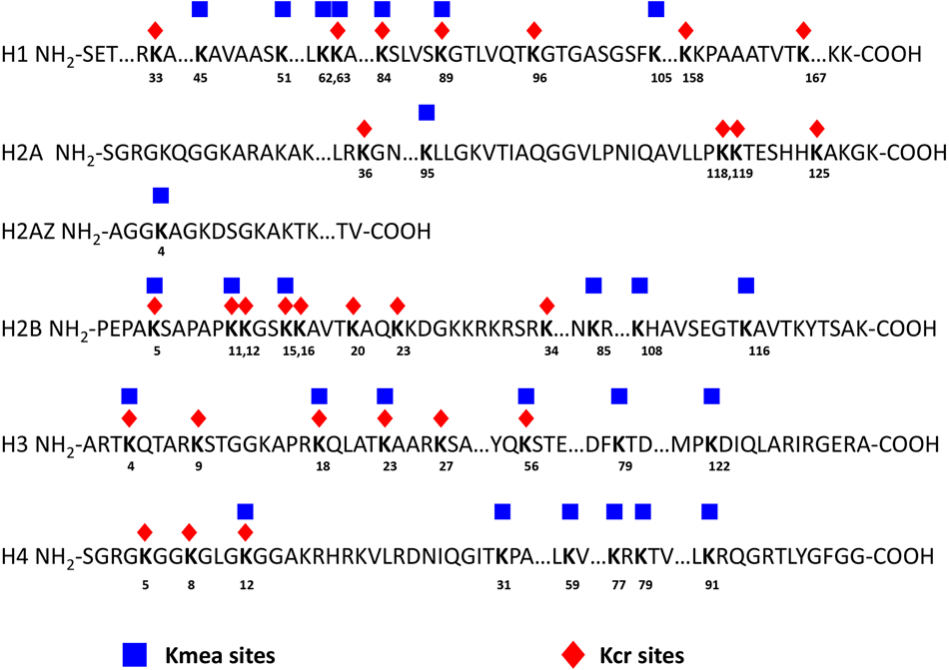
Maps of the identified Kmea and Kcr on HeLa histones. Schematic of the sequences of the 5 core histone proteins and 1 variant histone sequence (H2AZ) with sites of Kmea and Kcr identified in HeLa cells. The key for the modifications is as follows: Kmea, blue squares; Kcr, orange diamonds. Kmea sites include both those detected by IP-HPLC/MS/MS with pan Kmea antibody as well as sites that were validated by co-elution or ozonolysis in HeLa cells. Kcr sites were detected in our previous publication using HeLa cell histones^11^.

An understanding of the potential roles of Kmea will require that we know the modified lysine residues on histones. To identify other Kmea marks on core histones, we treated cells with sodium methacrylate for 24 h before harvesting cells and performing acid extraction. The resulting core histones were tryptically digested and enriched by immunoprecipitation with the pan anti-Kmea antibody. We performed HPLC-MS/MS analysis on the enriched sample and then analyzed the results using MASCOT software to search for lysine residues bearing +68.023 Da additional mass. For spectra with a MASCOT score greater than 20, we further manually evaluated spectral quality and removed low quality hits (Supplementary Fig. S7). Altogether, we identified a total 27 Kmea sites across the core histones from HeLa cells (Fig. 4). The positions of Kcr sites that we identified in our original screen of HeLa cells are shown for comparison^11^. A comparison between histone Kmea and Kcr suggest that more Kmea marks were detected outside N-terminal tails on histone H3, H4 and H2B. In addition, more Kcr sites were detected on histone H1 and H2A. These results demonstrate the widespread occurrences of histone Kmea and suggest the existence of possible distinct mechanisms directing histone lysine methacrylation and histone lysine crotonylation.

### HAT1 is a writer for Kmea

**Fig. 5:**
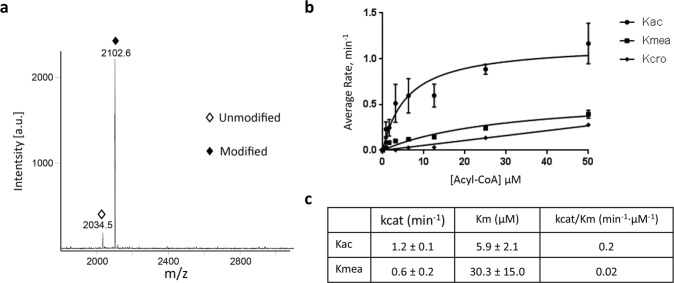
HAT1 catalyzes Kmea addition in vitro. **a** Synthetic peptides consisting of the first 20 amino acid residues of the H4 histone, H4 (1-20), were incubated with methacrylyl-CoA and recombinant HAT1 in vitro. MALDI-TOF analysis detected unmodified (open diamond) and methacrylated (solid diamond) peptides. **b** HAT1 was incubated with acetyl-CoA, methacrylyl- CoA, or crotonyl-CoA at varied concentrations and H4 (1-20) peptide substrates for 15 min, respectively. The enzymatic reaction was quantified using 7-diethylamino-3-(4’-maleimidylphenyl)-4-methylcoumarin (CPM) assay. **c** The reaction rate-acyl-CoA concentration was plotted with the Michaelis-Menten equation to get the kinetic constants Km and kcat for acetyl-CoA and methacrylyl-CoA substrates, respectively. The kcat/Km values were used to evaluate lysine acylation efficiency.

We hypothesized that previously classified acetyltransferases might be able to catalyze the addition of the methacryl moiety to lysine. This was based upon the evidence that several of the newly discovered lysine acylations are regulated by enzymes previously classified as acetyltransferases^4^. For example, lysine crotonylation and butyrylation can be catalyzed by p300^10,26,29^. We synthesized methacrylyl-CoA and screened for methacryltranferases. There are four major acetyltransferase families in mammals which include: HAT1, GCN5/PCAF, p300/CBP, and MYST. We selected MOF, GCN5, HAT1, and p300, to identify in vitro writers for Kmea, on the basis of being representative members of each of the four major acetyltransferase subfamilies. As expected, the four known acetyltransferases can catalyze in vitro Kac reaction when a synthetic histone H3 or H4 peptide was used as a substrate (Supplementary Figs. S8, S9). However, only recombinant HAT1 was able to catalyze the formation of Kmea in the presence of methacrylyl-CoA (Fig. 5a). We further analyzed the kinetics of HAT1 acetyl/acyl-transferase activity using methacrylyl-CoA, acetyl-CoA, and crotonyl-CoA as substrates (Fig. 5b). We found that the kcat/Km value for HAT1 with methacrylyl-CoA is 10-fold lower than the value for acetyl-CoA (Fig. 5c). We were unable to measure the kinetics of HAT1 for crotonyl-CoA beyond the slope of the kinetic curve, which is 0.0057 by linear regression. These data provide the first evidence that the addition of Kmea can be enzymatically catalyzed in vitro.

**Fig. 6:**
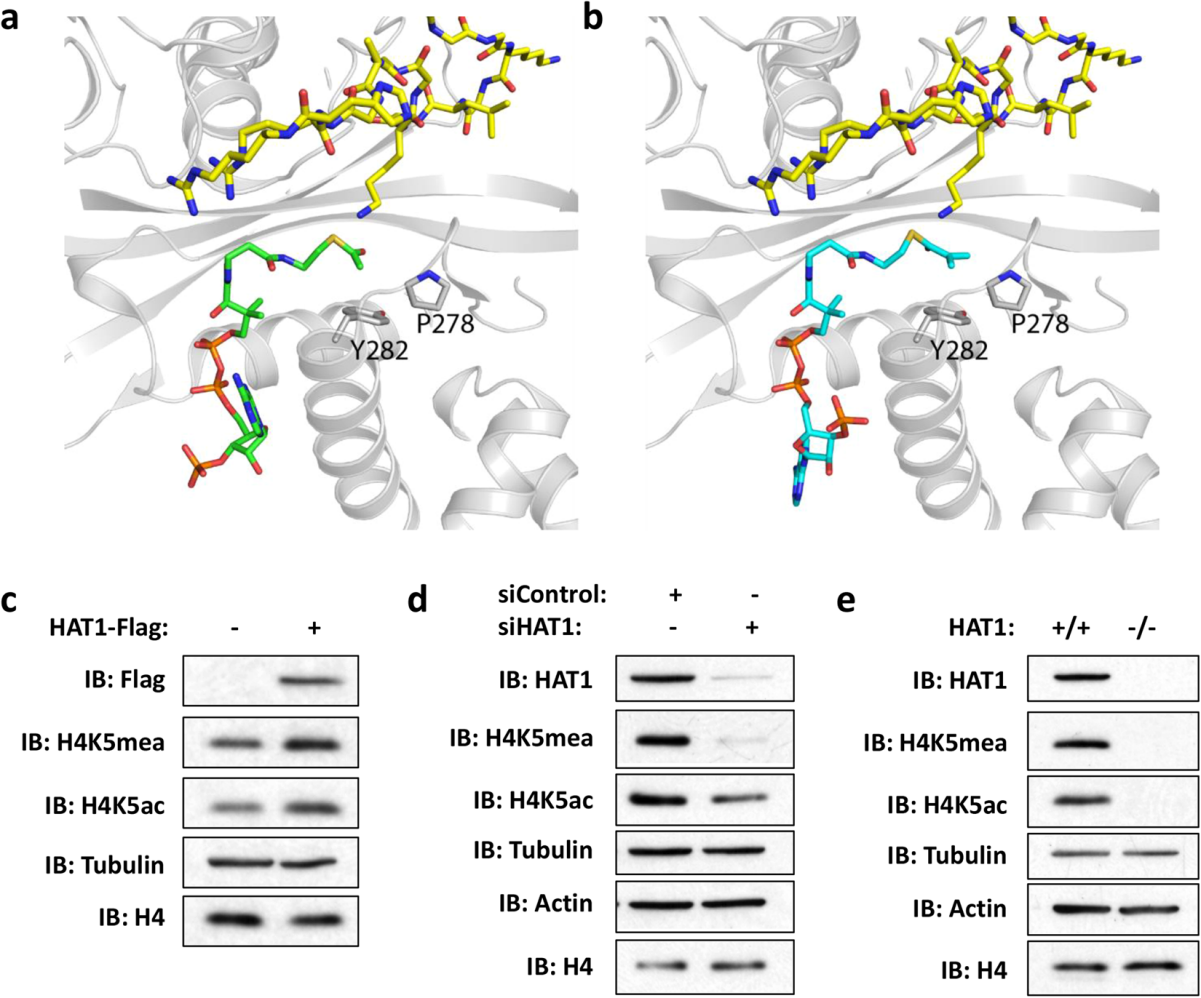
HAT1 regulates H4K5mea in cells. **a** Close-up view of the HAT1-acetyl-CoA interaction site in the crystal structure of HAT1 in complex with acetyl-CoA and histone H4 peptide (PDB ID: 2P0W). HAT1 is shown in cartoon representation in gray, acetyl-CoA in green, and histone H4 in yellow. **b** Close-up view of the methacrylyl-CoA interaction site with HAT1 in a model generated by fitting the methacrylyl-CoA into the isobutyryl-CoA electron density map in the crystal structure of HAT1-isobutyryl-CoA (PDB ID: 6VO5) for illustration purposes. HAT1 is shown in cartoon representation in gray, methacrylyl-CoA in cyan and histone H4 in yellow. **c** HEK 293 T were transfected with empty pcDNA 3.1 vector or pcDNA 3.1 HAT1-Flag plasmid for 48 h before harvesting. Cell lysates were prepared using RIPA buffer and then analyzed by western blot. **d** HEK 293 T cells were transfected for 72 h with 25 nM of control or HAT1 siRNA. RIPA lysates were probed using indicated antibodies. **e** MEF HAT1^−/−^ and MEF HAT1^+/+^ cells were grown in culture before harvesting lysates. RIPA lysates were assessed by western blot as indicated.

HAT1 catalyzes lysine acetylation by positioning acetyl-CoA in proximity to lysine residues of histone H4 in its active site (Fig. 6a). We generated a model of HAT1 in complex with methacrylyl-CoA using the isobutyryl-CoA electron density map from the crystal structure of HAT1-isobutyryl-CoA (PDB ID: 6VO5) (Fig. 6b). Similar to the structure with acetyl-CoA, this structure shows the ε-amine of H4K12 is positioned in close proximity to the carbonyl-carbon of the methacrylyl-CoA thioester bond in the active site of HAT1. HAT1 is known to acetylate newly synthesized histone H4 on lysine residues 5 and 12^30^. To test the effects of HAT1 on Kmea in cells, we used our site-specific antibody for H4K5mea that presents high specificity (Supplementary Fig. S5a). It is known that the HAT1-dependent H4K5ac modification is rapidly removed following incorporation of H4 into chromatin^31,32^. We thus hypothesized that we should use newly synthesized histones to analyze Kmea to detect dynamic changes of histone Kmea. To this end, we prepared lysates using RIPA buffer as previously described to acquire samples enriched for newly synthesized and soluble histones^33^. We hypothesized that H4K5mea levels in RIPA-soluble histones would increase with overexpression of HAT1. Consistent with our hypothesis, both H4K5ac and H4K5mea signals increased following overexpression of HAT1-Flag protein in HEK 293 T cells (Fig. 6c). Similarly, knockdown of HAT1 by siRNA but not by control siRNA decreased both H4K5ac and H4K5mea signals (Fig. 6d). Finally, both H4K5mea and H4K5ac levels were much lower in MEF HAT1^−/−^ samples relative to HAT1^+/+^ control samples (Fig. 6e). Together, our results indicate that HAT1 catalyzes the transfer of methacryl moiety to histone H4 in vitro and in mammalian cells.

### SIRT2 is an eraser for Kmea in vitro and in cells

**Fig. 7:**
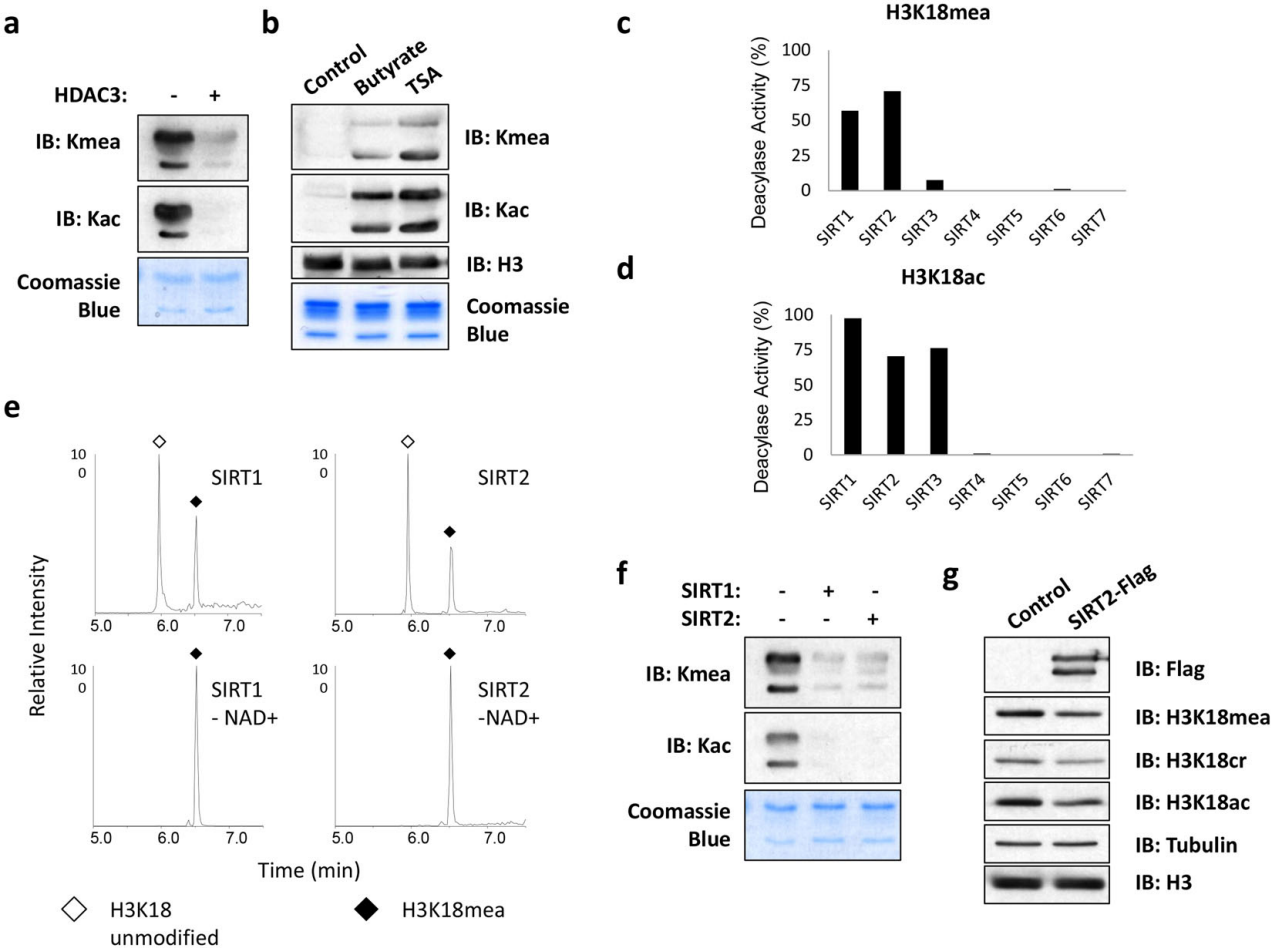
SIRT1 and SIRT2 remove methacryl from Kmea. **a** Acid extracted HeLa histones were incubated with or without recombinant HDAC3 for 12 h at 37 °C. Samples were then analyzed by western blot. **b** HEK 293 T cells were treated for 24 h with either 10 mM sodium butyrate or 1 μM TSA. The core histones were prepared by acid extraction method and then subjected to western blot analysis. **c**, **d** Quantitation of deacylase activity of SIRT 1-7 for in vitro screen using either H3K18mea peptide (c) or H3K18ac peptide (d) is shown. The deacylase activity percentage was calculated by dividing the area of the H3K18 unmodified chromatogram peak over the sum of the H3K18 unmodified and modified chromatogram peaks. **e** H3K18mea synthetic peptides were incubated with SIRT1 or SIRT2 in presence of reaction buffer with or without its cofactor NAD + . Chromatograms of each sample condition are shown. Open diamonds indicate the H3K18 unmodified peptide peaks. Filled diamonds indicate the H3K18mea peptide peaks. **f** Acid-extracted HeLa histones were incubated with or without recombinant SIRT1 or SIRT2 for 12 h at 37 °C. Samples were then analyzed by western blot. **g** HEK 293 T cells were transfected with either control empty pcDNA 3.1 vector or pcDNA 3.1 SIRT2-Flag. Whole cell lysates were prepared 48 h post-transfection and then subjected to western blot analysis with the indicated antibodies.

We and others have shown that many of the previously classified lysine deacetylases are able to enzymatically remove other types of lysine acylations in addition to lysine acetylation^34^. Crotonyl moieties on lysine residues can be enzymatically removed by HDAC1-3 and SIRT1-3^11–13,15^. A recent screen of HDAC1-11 that included previously discovered and hypothetical substrates bearing lysine acylations revealed that HDAC3 acts as an eraser for lysine methacrylation in vitro^35^. Consistent with this report, we observed that in vitro incubation of HDAC3 with histone extract significantly reduced the Kmea signal detected by western blot (Fig. 7a). Similarly, we observed a large increase in Kmea signal in cells treated for 24 h with the HDAC class I-II inhibitors butyrate and trichostatin A (Fig. 7b).

Unlike HDAC 1-11, the sirtuin family of enzymes has not been studied for the removal of methacryl from lysine residues. We screened recombinant SIRT1-7 for their ability to enzymatically remove methacryl moiety from synthetic H3K18mea peptides. We found that SIRT1 and SIRT2 had the strongest methacryllysine eraser activity in vitro (Fig. 7c). As a control, we used a peptide bearing H3K18ac, which was efficiently deacetylated by SIRT1-3 (Fig. 7d). We confirmed that the effects of SIRT1 and SIRT2 were due to their enzymatic activity as removal of the essential cofactor NAD + from the buffer abolished their ability to remove the methacryl moiety from peptide (Fig. 7e). Similar to our results using synthetic peptides, incubation of recombinant SIRT1 and SIRT2 enzymes with histone extract in vitro greatly reduced the Kmea western blot signal (Fig. 7f). These experiments confirm that SIRT1 and SIRT2 possess the ability to catalytically remove the methacryl moiety from lysines in vitro.

The sirtuin family of deacylases are reported to work on specific histone marks^36^. One of the SIRT2 substrates is H3K18ac, which has been reported to be regulated by the translocation of SIRT2 to the nucleus under certain conditions^37^. We hypothesized that we could also promote H3K18 deacylation following overexpression of SIRT2-Flag in HEK 293 T cells. We used a highly specific anti-H3K18mea antibody to test our hypothesis (Supplementary Fig. S5b). Consistent with our hypothesis, overexpression of SIRT2-Flag was associated with decreases in the H3K18ac, H3K18cr, and H3K18mea western blot signals relative to the empty vector control (Fig. 7g). In summary, these investigations identify SIRT2 as an enzyme capable of catalyzing the removal of H3K18mea both in vitro and in cells.

## Discussion

The landscape of histone posttranslational modifications has continued to expand in recent years. A particularly notable example is Kcr, an epigenetic modification that is associated with active transcription^10,11^. Kcr is associated with active gene expression during mouse spermatogenesis^11,22^. Similarly, Kcr is also associated with the de-novo activation of genes in RAW 264.7 macrophages following LPS stimulation^10^. Depletion of gut microbiota affects histone Kcr in the colon^21^. Kcr is also found widely on non-histone proteins, including ribosomal and myofilament proteins in zebrafish embryos^38^. Importantly, writers, readers, and erasers were identified that regulate histone Kcr^4^. MOF, p300, GCN5, and Esa1 are reported writers for Kcr on histones^10,14,39^. YEATs and PHD domain containing proteins have been identified as readers for Kcr on histones^17–20,40^. HDAC 1-3 and SIRT 1-3 are the erasers for Kcr^11–13,15^. Altogether, these data suggest that histone crotonylation is a crucial enzymatically regulated epigenetic modification. However, Kcr is not the only histone lysine acylation with functions in epigenetic signaling discovered in recent years. As another example, lysine succinylation (Ksucc) occurs on histones and is also associated with transcriptional regulation^41–43^.

Here we report the discovery of Kmea, a structural isomer of Kcr. Certain evidence presented here suggests that Kcr and Kmea could be connected to different regulatory circuits. For example, crotonyl-CoA and methacrylyl-CoA are derived from distinct metabolic pathways. Crotonyl-CoA is an intermediate product of β-oxidation of fatty acids as well as the catabolism of lysine and tryptophan^44^. In contrast, methacrylyl-CoA is an intermediate product in the mitochondrial catabolism of valine^45^. These differences imply that the accumulation and the regulation of cellular histone/protein Kcr and Kmea levels likely occur through distinct upstream metabolic pathways. An important unsolved issue concerning not only Kcr and Kmea, but also various histone acylations, is whether different metabolic pathways leading to generation of the corresponding CoA derivatives, would also ensure different gene regulatory functions. A noticeable difference between histone acetylation and acylation is their ability to modulate the binding of bromodomains, especially, the members of BET family to chromatin^29^. Histone methacrylation very likely belongs to the category of short chain histone acylations that could, along with the other acylations, create a barrier against bromodomain factor binding to chromatin. A published work on the role of histone butyrylation showed that the important feature of the highly active gene is not acetylation or butyrylation alone, but is the alternative and dynamic histone acetylation-butyrylation, which is the true signature of active transcription^29^. Histone methacrylation increases therefore the arsenal of chromatin regulatory circuits linking histone acylation to increasing numbers of metabolic pathways. Therefore, this new finding suggests that chromatin could be maintained in an active state depending on a larger number of metabolic pathways than previously anticipated.

The identification of enzymatic writers is essential for deciphering functional relevance of a new type of histone mark and signaling through lysine acylation. For example, identification of the first acetyltransferases represented a milestone for studying signaling pathways involving histone Kac marks^30,46,47^. The transcriptional activator p300 uses not only acetyl-CoA but also the newly discovered acyl-CoAs to modify histones and activate transcription in vitro^9,10,27,29^. In this work, we identify HAT1 as a writer for Kmea. The functional roles of HAT1 remain an area of active study. Recent evidence suggests that HAT1 regulates cellular metabolism. Mice that are heterozygous for HAT1 expression have defects in their mitochondria and present early aging phenotypes^48^. Another study found that HAT1 phosphorylation by AMPK promoted mitochondrial biogenesis^49^. Indeed, the HAT1-mediated acetylation of histones has been proposed to act as a nutrient sensor coupling the state of cellular metabolism to signals for cell division^33^. We speculate that the ability of HAT1 to use alternative acyl-CoAs like methacrylyl-CoA may provide additional ways for this enzyme to sense and respond to the state of cellular metabolism. This possibility is reminiscent of our previous finding showing that p300 uses different acyl-CoAs for the regulation of distinct pathways^27^. As such, the discovery of Kmea may provide new insight into deciphering the functions of HAT1 in future studies.

Inherited mutations in the ECHS1 and HIBCH enzymes of the valine catabolic pathway lead to the development of Leigh syndrome, a neurological disorder characterized by mitochondrial defects^50–52^. Methacrylyl-CoA accumulates in patients with these mutations and has been hypothesized to act as a causal factor in the disease through its electrophilic capacity to alkylate cysteines^50^. The discovery of Kmea raises a potential alternative mechanism for pathological effects of methacrylyl-CoA accumulation. In other in-born-errors of metabolism and physiological changes (e.g., diabetes and hypoxia), elevated levels of acyl-CoAs, such as butyryl-CoA, β-hydroxybutyryl-CoA, and lactyl-CoA, correlate with higher levels of lysine acylations^9,53–55^. An overabundance of certain lysine acylations is reported to have negative effects on ATP synthase activity, albeit to a relatively small degree^56^. Future studies are needed to determine the targets of Kmea modifications on non-histone proteins as well as their impact on chromatin-independent function.

Both spontaneous lysine acylation and enzyme-catalyzed lysine acylation reactions can occur. Extensive literature on the studies of epigenetic function of histone lysine acetylation and its regulation by acetyltransferases, e.g., p300, argue strongly that enzyme catalyzed histone acetylation is the driving force for histone acetylation. It was also reported that a regulatory enzyme is critical for function of histone Ksucc and Kcr marks^10,41^. These lines of evidence suggest that in nuclei, enzyme-catalyzed reaction instead of spontaneous reaction is largely responsible for the detected function of histone lysine acylations. Nevertheless, it is also known that acyl-CoAs can react nonenzymatically with the lysine’s amine group. The reaction rate is especially higher in the inner mitochondrial matrix where the pH is higher^57^. Thus, it is highly likely that spontaneous Kmea reactions happen in mitochondria.

While methacrylyl-CoA can be produced in mitochondria through valine catabolism, how methacrylyl-CoA accumulates in the nucleus is another subject that requires further study. Series of investigations have suggested that acetyl-CoA often needs to be produced within the nucleus to be incorporated into nuclear histones^58^. Similarly, nuclear α-KGDH complexes have been shown to provide succinyl-CoA for histone Ksucc^41^. We previously reported that knockdown of the nuclear and cytoplasmic enzyme ACSS2 is associated with depletion of histone Kcr^4^. In addition, the nuclear pools of crotonyl-CoA can be further metabolized into β- hydroxybutyryl-CoA by nuclear CDYL^22^. Whether similar enzymes regulate nuclear methacrylyl- CoA pools is still to be determined. Identification of Kmea and its regulatory elements open a new window to study cross talk between metabolism and epigenetic regulations.

## Materials and methods

### Reagents

We developed the pan anti-Kmea, anti-H3K18mea, and anti-H4K5mea antibodies in collaboration with PTM Bio Inc. (Chicago, IL). We obtained pan anti-Kcr (PTM-501) and pan anti-Kac (PTM-101) antibodies from PTM Bio Inc. (Chicago, IL). The anti-tubulin (ab6160), anti- H4 (ab31830) and anti-H3 antibody (ab12079) were obtained from Abcam (Cambridge, MA). Anti-Flag (F7425) was obtained from Sigma-Aldrich (St. Louis, MO). We obtained anti-H4K5ac antibody (39700) from Active Motif (Carlsbad, CA). The anti-actin antibody (4967 S) was from Cell Signaling Technologies (Danvers, MA). Methacrylic acid and crotonic acid were obtained from Sigma-Aldrich (St. Louis, MO). The d7-methacrylic acid was obtained from CDN Isotopes (Pointe-Claire, Quebec, Canada). Pooled siRNA for control (D-001810-10-05) and HAT1 (L- 011490-00-0005) were obtained from Horizon Discovery (Waterbeach, United Kingdom). Sequencing grade modified trypsin was obtained from Promega (Madison, WI). Chemicals used in buffers were obtained from Sigma-Aldrich (St. Louis, MO) unless otherwise noted.

### Cell culture

HeLa and HEK 293 T cells were obtained from ATCC and not validated further. MEF HAT1 wild type and knockout cells were prepared as previously published^31^. Cells were grown in high glucose DMEM (Thermo Fisher Scientific Inc., Waltham, MA) with 10% FBS at 37 °C in 5% CO_2_.

### Methacrylate and crotonate treatment

The salt forms of methacrylic acid and crotonic acid were prepared by raising pH to 7.0–7.3 using NaOH. HeLa cells were treated crotonate after reaching approximately 50% confluency. Cells were harvested for histone extraction at 24 h post treatment. HeLa cells treated with 20 mM d7-methacrylic acid (CDN Isotopes, Pointe-Claire, Quebec, Canada) were prepared in the same way.

### Plasmid transfection

HEK 293 T cells were grown to approximately 80% confluence before transfecting with 2 μg of plasmid DNA using Lipofectamine 3000 (Thermo Fischer Scientific Inc., Waltham, MA) according to manufacturer instructions. Culture media was replaced with fresh media at 24 h post-transfection. Cells were harvested by whole cell lysis at 48 h post-transfection.

### RNAi transfection

HEK 293 T cells were transfected with siRNA using Lipofectamine RNAiMAX (Thermo Fisher Scientific Inc., Waltham, MA) for reverse transfection according to manufacturer instructions. Briefly, we added 62.5 pmol of either siControl (Horizon Discovery plc, Waterbeach, United Kingdom, D-001810-10-05) or HAT1 (Horizon Discovery plc, Waterbeach, United Kingdom, L- 011490-00-0005) to 500 μL Opti-MEM (Thermo Fisher Scientific, Waltham, MA) per well of 6 well plates. Then 10.5 μL of RNAiMAX was added to each well. This mixture was incubated at room temperature for 20 min. Then 2 mL of HEK 293 T cell suspension in complete media was added to each well. The cells were incubated for 72 h post-transfection at 37 °C in 5% CO_2_. We replaced the media at the 24 and 48 h time points post-transfection with fresh complete media. Cells were then harvested and lysates were prepared using RIPA buffer.

### Preparation of cell lysates

#### Protein whole-cell lysate

Cells were harvested upon reaching experimental time points and were greater than 80% confluent. Cells were washed in PBS once before the addition of lysis buffer (60 mM Tris HCL, pH 6.8; 10% glycerol; 2% SDS; and 5% β-mercaptoethanol) with the additional inhibitors 1 mM PMSF, 10 mM nicotinamide, and 5 mM sodium butyrate. The adherent cell lysate was scraped from dish and transferred to a microcentrifuge tube. The lysate was then briefly vortexed, boiled for 5 min, and centrifuged at greater than 16,000× *g* for 5 min before supernatant was transferred to new tube.

#### RIPA lysate

For experiments involving HAT1 validation, an alternative lysis approach was used to enrich nascent histones as previously described^33^. Briefly, cells were washed in PBS once before the addition of RIPA buffer (50 mM Tris-HCL, pH 7.4; 150 mM NaCl; 0.1% SDS; 0.5% sodium deoxycholate; 1% Triton X-100) with the additional inhibitors 1 mM PMSF, 10 mM nicotinamide, and 5 mM sodium butyrate. Adherent cells were scraped in lysis buffer using cell scraper. Lysates were briefly vortexed before centrifuging at greater than 16,000× *g* for 10 min Then supernatant was transferred to a new tube. Laemmli buffer was added to samples that were boiled for 5 min prior to use in SDS-PAGE.

### Histone extraction

Acid extraction of histones was performed according to modified version of a published protocol^59^. Briefly, cells were harvested in 10-cm plates after reaching approximately 90% confluency. Cells were washed in phosphate buffered saline (PBS) before detaching from plate by cell scraper. Cells were then washed and pelleted in PBS twice at 1000× *g* for 5 min at 4 °C. Cellular plasma membranes were lysed in extraction buffer (10 mM HEPES pH 7.0, 10 mM KCl, 1.5 mM MgCl_2_, 0.34 M sucrose, 0.5% NP-40, 5 mM sodium butyrate, 10 mM nicotinamide) for 10 min with gentle agitation at 4 °C. Nuclei were then pelleted at 2000× *g* at 4 °C for 10 min and supernatant was discarded. Cells were resuspended in extraction buffer without NP-40, cell nuclei were pelleted at 2000× *g* at 4 °C for 5 min. Nuclear pellets were then lysed in a nuclear lysis buffer (3 mM EDTA, 0.2 mM EGTA, 5 mM sodium butyrate, 10 mM nicotinamide) for 30 min with gentle agitation at 4 °C. Chromatin was pelleted at 6500× *g* for 5 min at 4 °C and then resuspended in 0.4 N H2SO4. Chromatin suspension in acid was incubated overnight at 4 °C with gentle agitation. After centrifuging at 16,000× *g* for 10 min, acid soluble supernatant was collected and used in trichlorooacetic acid (TCA) precipitation. Following TCA precipitation, histones were pelleted and washed three times in cold acetone. Histones were finally resuspended in deionized water.

### Trypsin digestion

Isolated histones were in-solution digested using sequencing grade modified trypsin at a ratio of 50:1, respectively. Buffer was adjusted to pH 8 using ammonium bicarbonate. Samples were incubated at 37 °C overnight. Following incubation, samples were boiled for 1 min to inactivate trypsin.

### Peptide enrichment by immunoprecipitation and identification by HPLC/MS/MS

We began by conjugating pan anti-Kmea antibodies to nProtein A Sepharose beads (GE Healthcare Bio-Sciences Corp., Piscataway, NJ). The bead conjugated antibodies were then incubated in the presence of tryptically digested peptides at 4 °C with gentle agitation. We washed the beads with NETN buffer (50 mM Tris-Cl pH 8.0, 1 mM EDTA, 100 mM NaCl, 0.5% NP-40) three times. This was followed by two washes in ETN buffer which lacked the 0.5% NP- 40. This was followed once with deionized water. We then eluted peptides using 0.1% trifluoroacetic acid. The eluted peptides were then dried in a SpeedVac (Thermo Fisher Scientific Inc.).

Dried peptides samples were dissolved in HPLC Buffer A (0.1% formic acid in water, v/v) and loaded into a homemade capillary column (10-cm length × 75-mm ID, 3-μm particle size, Dr. Maisch GmbH, Ammerbuch, Germany) attached to an EASY-nLC 1000 system (Thermo Fisher Scientific Inc., Waltham, MA). We separated and eluted peptides along a gradient of 2 to 90% HPLC Buffer B (0.1% formic acid in acetonitrile, v/v) in HPLC Buffer A at a flow rate of 200 nL min^−1^ over 60 min. The peptides were ionized and analyzed using a Q-Exactive mass spectrometer (Thermo Fisher Scientific Inc., Waltham, MA).

### Validation of methacryllysine peptides using HPLC/MS/MS

We injected synthetic methacrylated or crotonylated peptides, with or without mixing with tryptically digested histones derived from cultured cells, into nano-HPLC system that is online coupled with a LTQ Orbitrap Velos Mass Spectrometer. The HPLC/MS/MS analysis were carried out as described above.

### Dot blot assay

Synthetic peptides were spotted onto nitrocellulose membrane in the amounts indicated in the figures. Membranes were incubated in blocking buffer (3% bovine serum albumin, 20 mM tris- buffered saline pH 7.6, 0.1% Tween-20) for 1 h at room temperature with gentle agitation. Membranes were incubated in solution of primary antibody diluted in 1% BSA blocking buffer and incubated for 30 min at room temperature with gentle agitation. Blots were washed three times in TBST. Then, membranes were incubated in secondary antibody diluted in 1% BSA in TBST for 30 min at room temperature with gentle agitation. Blots were washed three times in TBST and then incubated for 1 min in enhanced chemiluminescence reagent. Film was exposed in dark room to visualize antibody binding.

### Western blot and peptide competition assay

The protein samples of interest were resolved in SDS-PAGE and transferred to 0.2 μm PVDF membranes. Membranes were incubated in blocking buffer (3% bovine serum albumin, 20 mM tris-buffered saline pH 7.6, 0.1% Tween-20) for 1 h at room temperature. Membranes were probed with primary antibody in 1% BSA blocking buffer overnight at 4 °C with gentle agitation. Secondary HRP conjugated antibodies were incubated for 1 h at room temperature. Membranes were washed 3 times for 8 min each in TBST (20 mM tris-buffered saline pH 7.6, 0.1% Tween-20) following both primary and secondary antibody incubations. Film was exposed in dark room after incubating for 1 min in enhanced chemiluminescence reagent with gentle agitation.

For peptide competition assay, a pan anti-Kmea antibody was incubated in the presence of 500 molar excess of peptide libraries for 4 h at room temperature with gentle incubation. This solution was used as the primary antibody incubation overnight at 4 °C with gentle agitation. All other steps are consistent with the western blot protocol as described.

### Expression and purification of HAT1

The expression and purification of human HAT1 (20-341) was carried out following the method described in Hong Wu et al.^60^. The pET28a-LIC-HAT1 plasmid (Addgene, plasmid #25239) was transformed into BL21 (DE3)/RIL competent cells through heat-shock and then spread on plates containing antibiotics kanamycin and chloramphenicol. Protein expression was induced by the addition of IPTG (final concentration: 1.0 mM) and shaken for 16 h at 16 °C. The cells were collected and suspended in lysis buffer (50 mM Na-phosphate (pH 7.4), 250 mM NaCl, 5 mM imidazole, 5% glycerol, 2 mM β-mercaptoethanol, and 1 mM PMSF) then disrupted using the cell disruptor. The supernatant was passed through a column containing Ni-NTA resin equilibrated with column washing buffer (20 mM Tris-HCl (pH 8.0), 250 mM NaCl, 5% glycerol, 30 mM imidazole, and 1 mM PMSF) and the resin was washed twice with the same column washing buffer. Next, the resin was washed three times with buffer containing a higher concentration of imidazole (20 mM Tris-HCl (pH 8.0), 250 mM NaCl, 5% glycerol, 50 mM imidazole, and 1 mM PMSF). The resin then was eluted with elution buffer (20 mM Tris-HCl (pH 8.0), 250 mM NaCl, 5% glycerol, 500 mM imidazole, and 1 mM PMSF), and dialyzed in the dialysis buffer (25 mM Tris-HCl (pH 8.0), 150 mM NaCl, 10% glycerol, 1 mM DTT) at 4 °C overnight. Thrombin was added to the dialyzed protein containing HAT1 and dialyzed in thrombin cleavage buffer (20 mM Tris-HCl (pH 8.0), 100 mM NaCl, 2.5 mM CaCl_2_, 5% glycerol, 1 mM DTT) for 20 h at 4 °C to remove the His6×-tag. The resultant protein was concentrated and purified by anion exchange chromatography using the NGC FPLC system. The identification of HAT1 was confirmed using SDS-PAGE. Millipore centrifugal filter and Bradford assay were used to concentrate and determine the protein’s concentration, respectively. Lastly, the protein was aliquoted, flash frozen by liquid nitrogen and stored at −80 °C.

### Synthesis of methacrylyl-CoA

CoA (5 mg, 0.0065 mmol) was dissolved in 1.5 mL of 0.5 M NaHCO3 (pH 8.0) and cooled down on ice bath. Then methacrylic anhydride (10.02 mg, 0.065 mmol) in 1 mL of acetonitrile/acetone (1:1 v/v) was added dropwise to the CoA solution. The reaction solution was stirred at 4 °C overnight, and then quenched by adjusting pH to 4 with 1 M HCl. The reaction mixture was subjected to RP-HPLC purification with gradient 5%–45% acetonitrile over 30 min at flow rate 5 mL/min; UV wavelengths were set at 214 and 254 nm for peak detection. HPLC eluents were 0.05% TFA in water (solution A) and 0.05% TFA in acetonitrile (solution B). The fractions were collected and lyophilized after flash-freeze with liquid nitrogen. Methacrylyl-CoA was collected as white solid, 3.6 mg, yield 72%. 1H NMR (400 MHz, D2O) δ 8.54 (s, 1H), 8.32 (s, 1H), 6.10 (s, 1H), 5.96 (s, 1H), 5.58 (s, 1H), 4.49 (s, 1H), 4.16 (s, 2H), 3.91 (s, 1H), 3.76 (dd, J = 9.5, 4.4 Hz, 1H), 3.50 (d, J = 5.0 Hz, 1H), 3.33 (t, J = 6.3 Hz, 2H), 3.23 (dd, J = 11.8, 5.6 Hz, 2H), 2.92 (t, J = 6.0 Hz, 2H), 2.32 (t, J = 6.3 Hz, 2H), 1.78 (s, 3H), 0.83 (s, 3H), 0.70 (s, 3H).

### In vitro acetyltransferase assay

Acetyl-CoA lithium salt (A2181) was purchased from Sigma-Aldrich (St. Louis, MO) and dissolved in water to make the stock solution. The thiol-reactive probe 7-diethylamino-3-(4′-maleimidylphenyl)-4-methylcoumarin (CPM) (Cat# D346, Thermo Fischer Scientific Inc., Waltham, MA) was dissolved in 100% DMSO to make the stock solution at 1 mM. Both solutions were aliquoted, stored at −20 °C and ready to use immediately before experiments. We combined 0.5 μM recombinant enzyme, 100 μM acetyl-CoA or methacrylyl-CoA, with 50 μM histone H3 or H4 peptide. Reaction mixtures were incubated at 30 °C for 2 h. Synthetic histone peptides substrates consisted of the first 20 amino acids of the N-terminal tails of histones H3 and H4 with acetylation of the N-terminal peptide amine. The sequences for H3 (1- 20) and H4 (1-20) were Ac-ARTKQTARKSTGGKAPRKQL and Ac- SGRGKGGKGLGKGGAKRHRK, respectively. Reactions with GCN5 and p300 as enzymes were paired with synthetic H3 (1-20) peptide as substrate. Similarly, reactions with MOF and HAT1 were paired with synthetic H4 (1-20) as substrate. Samples were analyzed by MALDI- TOF MS to identify if peptide substrates were modified in the reactions, respectively.

### Steady-state kinetics of HAT1 acetylation and methacrylation

Varied concentrations of acetyl-CoA (Sigma Aldrich, Cat# A2181), methacrylyl-CoA and crotonyl-CoA were incubated with 0.1 µM HAT1 and 200 µM of H4 (1–20) peptide (sequence: Ac-SGRGKGGKGLGKGGAKRHRK). The enzymatic reactions were conducted at 30 °C for 15 min in KAT reaction buffer containing 50 mM HEPES-Na and 0.1 mM EDTA-Na, pH 8.0. The reactions were quenched by 50% isopropanol and incubated with the probe 7-diethylamino-3-(4′-maleimidylphenyl)-4-methylcoumarin (abbr. CPM, Thermo Fisher, Cat# D346) in the darkness for 30 min at room temperature, producing the fluorescent CPM-SCoA complex. The fluorescence intensities were measured at an excitation of 392 nm and an emission of 482 nm with FlexStation®3 microplate reader to obtain reaction rate for each datapoint. Kinetic constants including binding affinity (Km) and catalytic rate (kcat) were determined by fitting the acyl-CoA concentration- rate curve to the Michaelis-Menten equation using GraphPad Prism.

### In vitro sirtuin peptide assay

Recombinant sirtuin proteins were obtained from BPS Biosciences Inc. (San Diego, CA). In vitro sirtuin reactions were performed by combining 0.5 μM synthetic peptide with 0.25 μM sirtuin in 20 mM Tris-HCL, pH 8 with 1 mM NAD + and 1 mM DTT in total volume of 50 μl. Mixtures incubated at 37 °C for 1 h. Reaction was quenched by adding 50 μl of 200 mM HCl and 320 mM acetic acid in HPLC grade methanol. Samples were dried using SpeedVac. Samples were desalted by ZipTip before being subjected to HPLC/MS/MS analysis. Percent deacetylase activity was measured by dividing the area of the unmodified peptide chromatographic peak over the sum of the area of the unmodified and modified chromatographic peaks.

### In vitro HDAC assay

Recombinant HDAC3, SIRT1, and SIRT2 were all obtained from BPS Biosciences Inc (San Diego, CA). The reaction buffer for the sirtuins was 20 mM Tris-HCL, pH 8 with 1 mM NAD + and 1 mM DTT. HDAC3 reaction buffer consisted of 25 mM Tris pH 8, 130 mM NaCl, 3.0 mM KCl, 1 mM MgCl_2_, and 0.1% PEG8000, pH 8.0. We treated HeLa cells with 20 mM sodium methacrylate for 24 h prior to performing histone extraction. The recombinant enzyme of interest was mixed with a histone extract at a ratio of 0.2 μg enzyme per 2 μg of histone extract in the appropriate buffer. Mixtures were incubated at 37 °C for 12 h. The samples were then analyzed by western blotting.

## Supplementary information

SI_after_proof
