# Supplementary material for: Histone lysine methacrylation is a dynamic post-translational modification regulated by HAT1 and SIRT2
Source: Cell Discov. 2021 Dec 28;7:122. doi: 10.1038/s41421-021-00344-4 (PMC8712513; doi:10.1038/s41421-021-00344-4)
Supplement: Supplementary file 1 — SI_after_proof [file 41421_2021_344_MOESM1_ESM.pdf]

Supplementary Information for

Histone lysine methacrylation is a dynamic post-translational modification regulated by HAT1 and SIRT2

Authors

Kyle Delaney[1], Minjia Tan[1, 7], Zhesi Zhu[2], Jinjun Gao[1], Lunzhi Dai[1, 8], Sunjoo Kim[3], Jun Ding[1], Maomao He[2], Levon Halabelian[4], Lu Yang[1], Prabakaran Nagarajan[5], Mark Robert Parthun[5], Sangkyu Lee[3], Saadi Khochbin[6], Yujun George Zheng[2], and Yingming Zhao[1]

Affiliations:

[1]Ben May Department for Cancer Research, The University of Chicago, Chicago, IL, 60637, USA

[2]Department of Pharmaceutical and Biomedical Sciences, University of Georgia, Athens, GA, USA.

[3]College of Pharmacy, Research Institute of Pharmaceutical Sciences, Kyungpook National University, Daegu, South Korea

[4]Structural Genomics Consortium, University of Toronto, Toronto, ON, M5G 1L7, Canada.

[5]Department of Biological Chemistry and Pharmacology, The Ohio State University, Columbus, OH, USA

[6]CNRS UMR 5309, INSERM, U1209, Université Grenoble Alpes, Institut Albert Bonniot, 38700 Grenoble, France.

[7]Current address: Shanghai Institute of Materia Medica, Chinese Academy of Sciences, 555 Zuchongzhi Road, Shanghai 201203, P.R. China.

[8] Current address: Department of General Practice, State Key Laboratory of Biotherapy, West China Hospital, Sichuan University, and Collaborative Innovation Center of Biotherapy, Chengdu, 610041, P.R. China

.

Correspondence: yingming.zhao@uchicago.edu

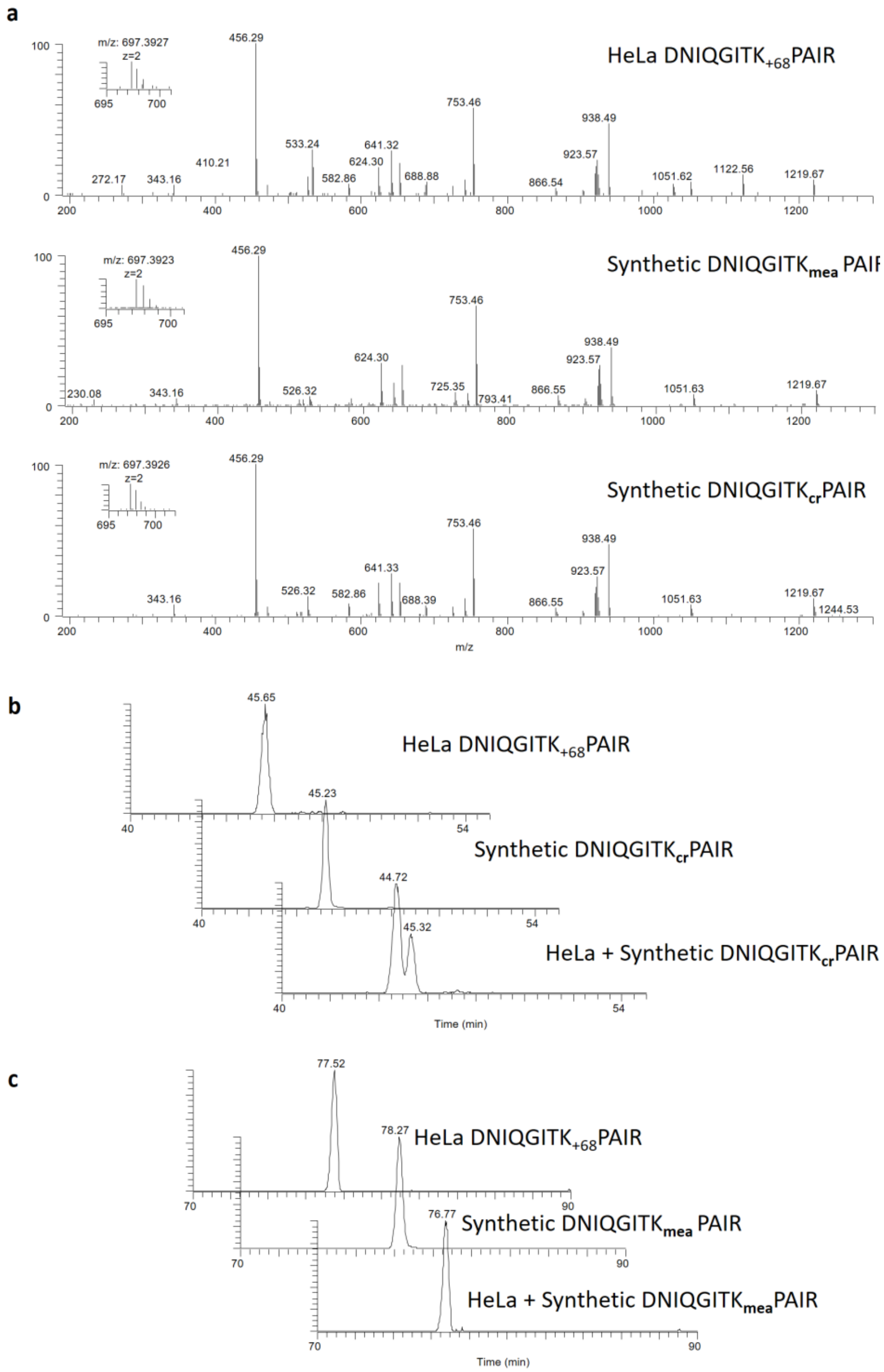

Supplementary Fig. S1: MS/MS and HPLC co-elution experiment to verify an H4K31mea, DNIQGITK+68PAIR. a MS/MS spectrum of HeLa histone peptide (top), synthetic H4K31mea

peptide (middle), and synthetic H4K31cr peptide (bottom). **b** Extraction ion chromatograms of peptide (DNIQGITK$_{+68}$PAIR) derived from HeLa histones (top), synthetic H4K31cr peptide (middle), and a mixture of both synthetic peptide with *in vivo* sample (bottom). **c** Extraction ion chromatograms of the peptide (DNIQGITK$_{+68}$PAIR) derived from HeLa histones (top), synthetic H4K31ma peptide (middle), and a mixture of both synthetic peptide with *in vivo* sample (bottom).

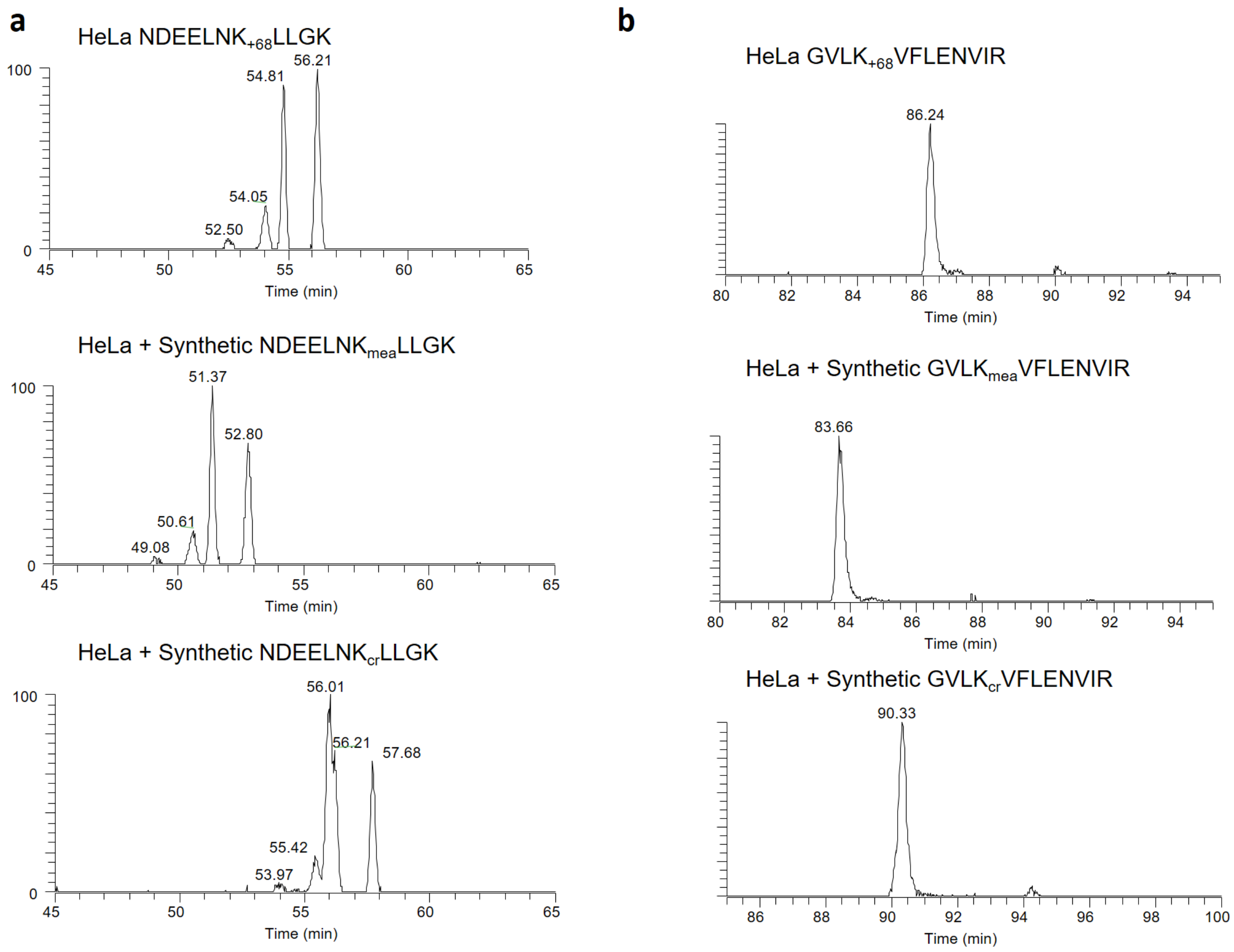

Supplementary Fig. S2: Co-elution of H2AK95mea and H4K59mea was insufficient for validation. a Extraction ion chromatograms of a peptide derived from HeLa histones (top), a mixture of synthetic peptide H2AK95mea (NDEELNK$_{mea}$LLGK) peptide with a peptide, NDEELNK$_{+68}$LLGK, derived from HeLa histones (middle), and a mixture of synthetic H2AK95cr (NDEELNK$_{cr}$LLGK) peptide with the NDEELNK$_{+68}$LLGK derived HeLa histones (bottom). **b** Extraction ion chromatograms of a peptide, GVLK$_{+68}$VFLENVIR, derived from HeLa histones (top), a mixture of synthetic peptide H4K59mea (GVLK$_{mea}$VFLENVIR) peptide with the peptide, GVLK$_{+68}$VFLENVIR, derived from HeLa histones HeLa sample (middle), and a mixture of synthetic H4K59cr (GVLK$_{cr}$VFLENVIR) peptide with the peptide, GVLK$_{+68}$VFLENVIR, derived from HeLa histones (bottom). Both Kmea and Kcr synthetic peptides co-eluted with peaks from HeLa sample.

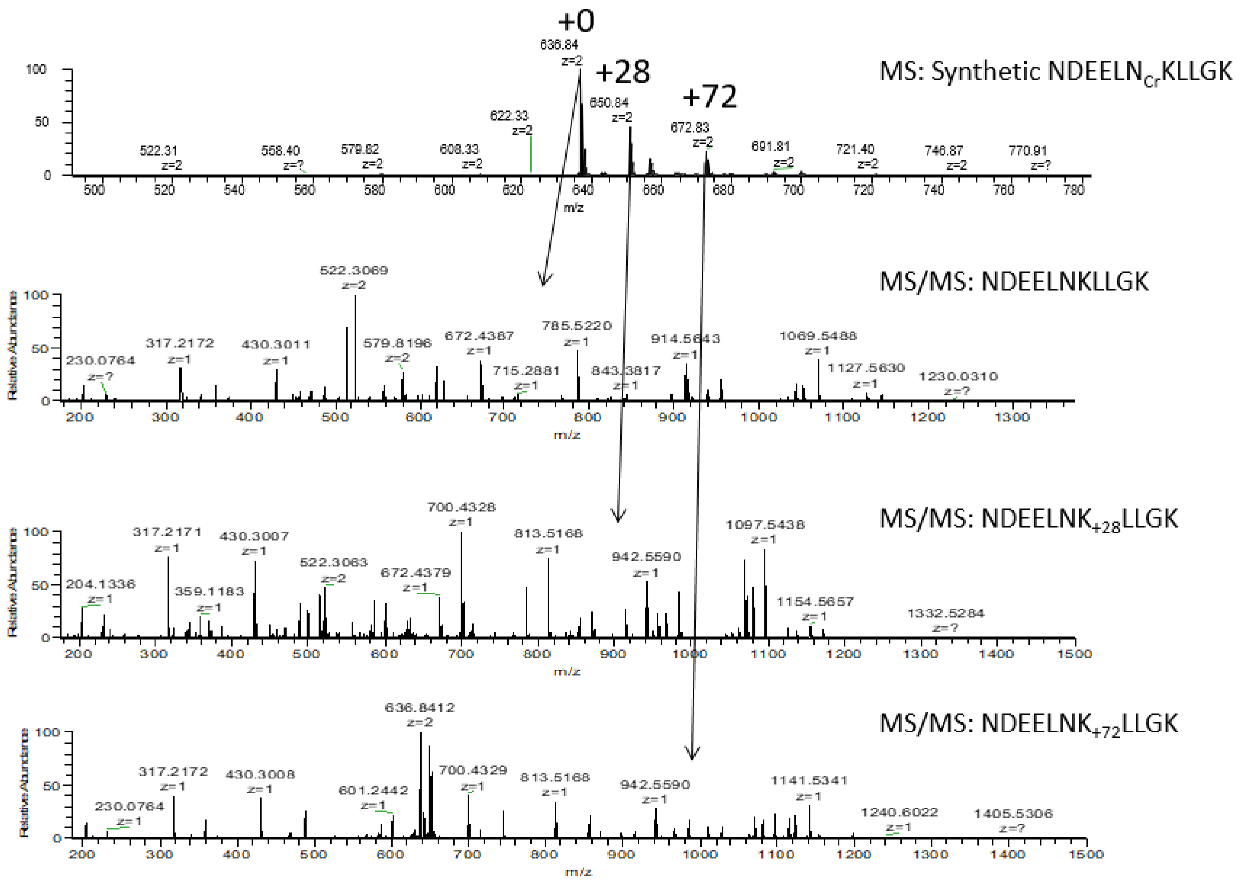

Supplementary Fig. S3: Ozonolysis of synthetic H2AK95cr peptide. Three peaks appeared in MS following O_3 oxidation of a synthetic H2AK95cr peptide, $NDEELNK_{cr}LLGK$ (top). These corresponded to the unmodified, +28 Da, and +72 Da mass shifted forms of the peptide. Both the $NDEELNK_{+28}LLGK$ and $NDEELNK_{+72}LLGK$ peptides corresponded to the expected products of O_3 oxidation of the crotonylated peptide. The MS/MS spectra of each of these peptides is shown as indicated.

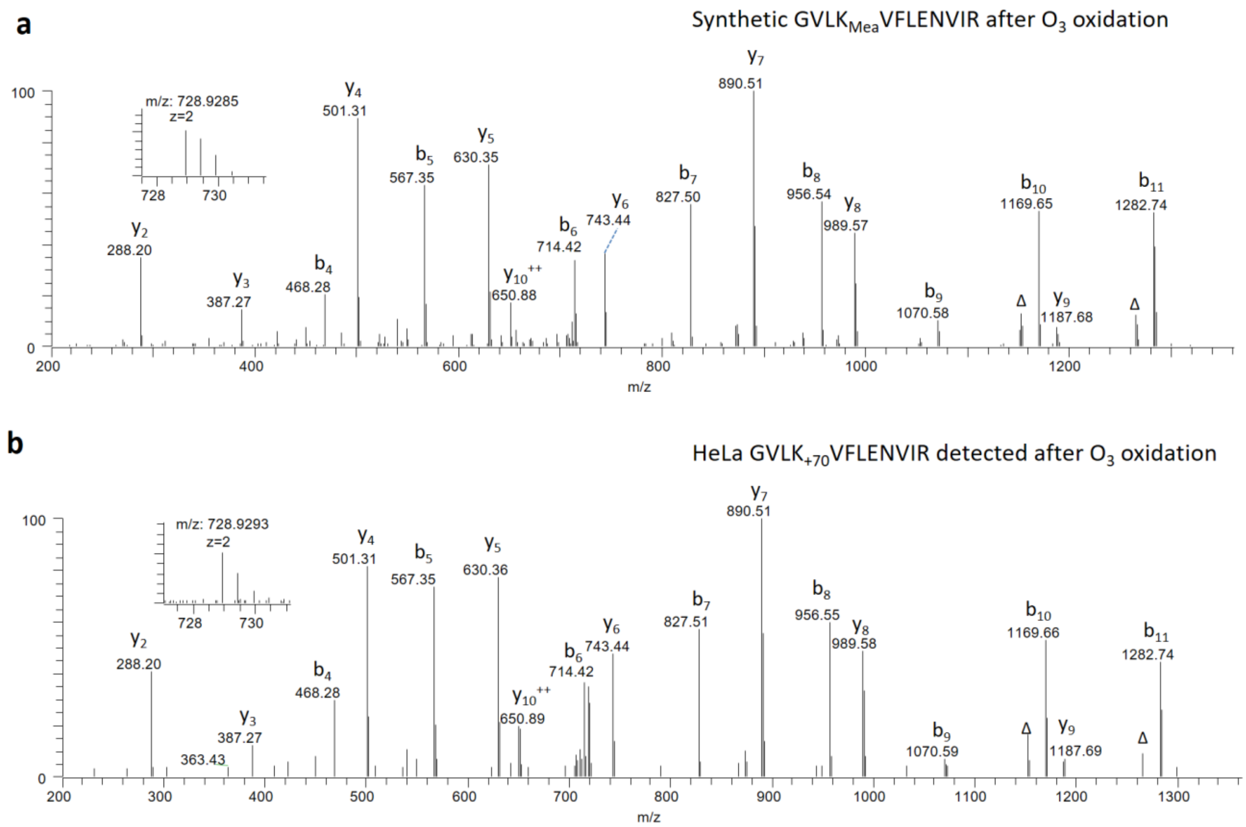

Supplementary Fig. S4: Validation of H4K59mea by ozonolysis. a MS/MS of GVLK$_{+70}$VFLENVIR peptide detected following O3 oxidation of a synthetic H2AK59mea peptide, GVLK$_{Mea}$VFLENVIR. **b** MS/MS of a GVLK$_{+70}$VFLENVIR peptide detected in O$_3$ oxidized peptides that were derived from HeLa histones.

a

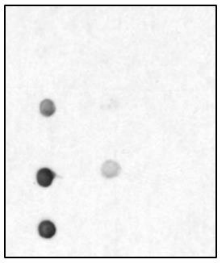

1 ng H4K5mea	64 ng H4K5ac	64 ng H4K5
4 ng H4K5mea	64 ng H4K5pr	
16 ng H4K5mea	64 ng H4K5bu	
64 ng H4K5mea	64 ng H4K5cr	

IB: H4K5mea

b

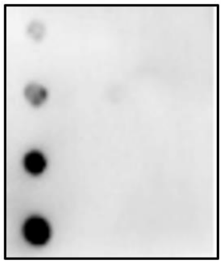

1 ng H3K18mea	64 ng H3K18ac	64 ng H3K9mea
4 ng H3K18mea	64 ng H3K18pr	64 ng H3K18
16 ng H3K18mea	64 ng H3K18b u	
64 ng H3K18mea	64 ng H3K18cr	

IB: H3K18mea

c

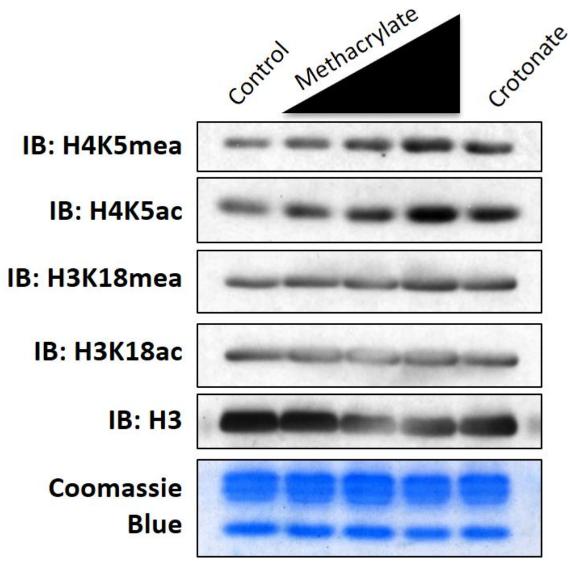

Supplementary Fig. S5: Validation of site-specific anti-Kmea antibodies. Dot blot assays were used to assess specificities of the pan anti-H4K5mea **a** and anti-H3K18mea **b** antibodies. The H4K5mea peptide has the sequence CSGRGK$_{mea}$GGKGLGK where Kmea is methacryllysine. The H3K18mea peptide has the sequence CTGGKAPRK$_{mea}$QLATKAA where Kmea is methacryllysine. The control peptides for both differ in modification status of the central lysine residue where the modifications are abbreviated as follows: ac, acetyl; pr, propionyl; bu, butyryl; and cr, crotonyl. A H3K9mea peptide with the sequence CRTKQTARK$_{mea}$STGGKAP was also used in testing anti-H3K18mea antibody specificity. The amount and identity of peptide spotted in each position of the membrane is indicated in the accompanying table (right). **c** HeLa cells were treated with 0, 1, 3, or 5 mM sodium methacrylate or 5 mM sodium crotonate for 24 hours. Histones were acid extracted and subjected to western blot analysis.

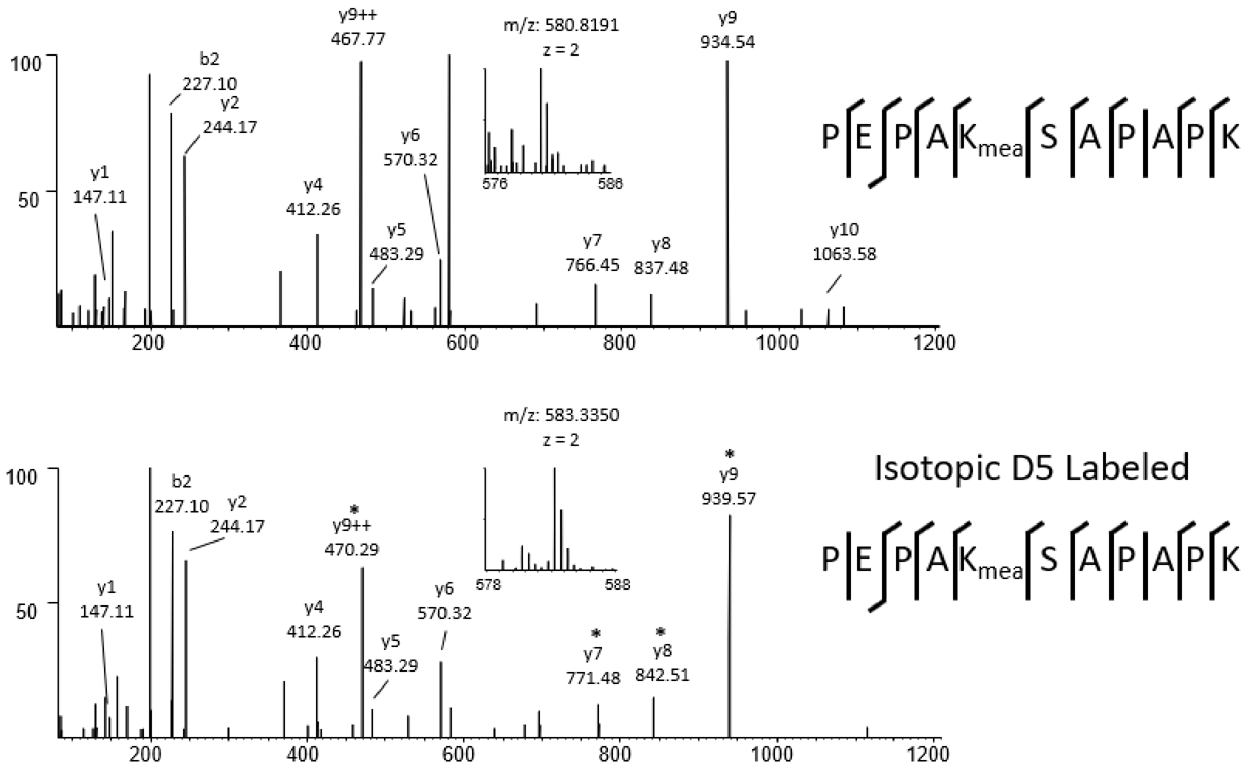

Supplementary Fig. S6: Deuterium labeled sodium methacrylate is directly incorporated into H2BK5mea. MS/MS spectra of H2BK5mea isolated from HeLa histones following treatment with methacrylate (top). MS/MS spectra of isotopic H2BK5mea isolated from HeLa histones following treatment with d-7 methacrylate (bottom). (*) indicate b and y ions with 5 Da mass shift consistent with incorporation of the isotopic metabolite.

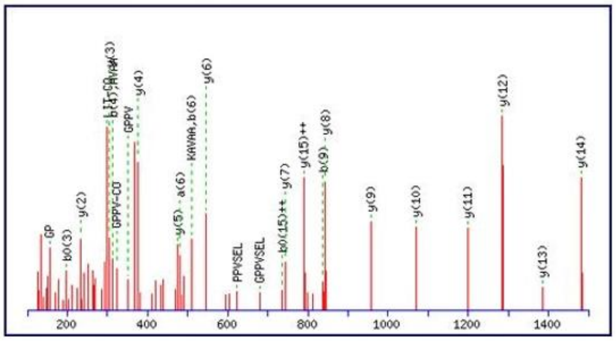

H1K45mea

MS/MS Fragmentation of **ASGPPVSELITKAVAASK**
Found in **P16402**, Histone H1.3

Match to Query 2445: 1792.987550 from(897.501051,2+)

Monoisotopic mass of neutral peptide Mr(calc): 1792.99
Variable modifications:
K12 : Methacryl (K)
Ions Score: 90 **Expect:** 1.1e-006
Matches (Bold Red): 28/408 fragment ions using 40 most intense peaks

#	Immon.	a	a++	a*	a*++	a0	a0++	b	b++	b*	b*++	b0	b0++	Seq.	y	y++	y*	y*++	y0	y0++	#
1	44.05	44.05	22.53					72.04	36.53					A							18
2	60.04	131.08	66.04			113.07	57.04	159.08	80.04			141.07	71.04	S	1722.96	861.99	1705.94	853.47	1704.95	852.98	17
3	30.03	188.10	94.56			170.09	85.55	216.10	108.55			**198.09**	99.55	G	1635.93	818.47	1618.90	809.96	1617.92	809.46	16
4	70.07	285.16	143.08			267.15	134.08	**313.15**	157.08			295.14	148.07	P	1578.91	**789.96**	1561.88	781.45	1560.90	780.95	15
5	70.07	382.21	191.61			364.20	182.60	410.20	205.61			392.19	196.60	P	**1481.86**	741.43	1464.83	732.92	1463.85	732.43	14
6	72.08	**481.28**	241.14			463.27	232.14	**509.27**	255.14			491.26	246.13	V	**1384.80**	692.91	1367.78	684.39	1366.79	683.90	13
7	60.04	568.31	284.66			550.30	275.65	596.30	298.66			578.29	289.65	S	**1285.74**	643.37	1268.71	634.86	1267.73	634.37	12
8	102.05	697.35	349.18			679.34	340.17	725.35	363.18			707.34	354.17	E	**1198.70**	599.86	1181.68	591.34	1180.69	590.85	11
9	86.10	810.44	405.72			792.43	396.72	**838.43**	419.72			820.42	410.71	L	**1069.66**	535.33	1052.63	526.82	1051.65	526.33	10
10	86.10	923.52	462.26			905.51	453.26	951.51	476.26			933.50	467.26	I	**956.58**	478.79	939.55	470.28	938.57	469.79	9
11	74.06	1024.57	512.79			1006.56	503.78	1052.56	526.78			1034.55	517.78	T	**843.49**	422.25	826.47	413.74	825.48	413.25	8
12	169.13	1220.69	610.85	1203.66	602.33	1202.68	601.84	1248.68	624.85	1231.66	616.33	1230.67	615.84	K	**742.45**	371.73	725.42	363.21	724.44	362.72	7
13	44.05	1291.73	646.37	1274.70	637.85	1273.71	637.36	1319.72	660.36	1302.69	651.85	1301.71	651.36	A	**546.32**	273.67	529.30	265.15	528.31	264.66	6
14	72.08	1390.79	695.90	1373.77	687.39	1372.78	686.90	1418.79	709.90	1401.76	701.38	1400.78	700.89	V	**475.29**	238.15	458.26	229.63	457.28	229.14	5
15	44.05	1461.83	731.42	1444.80	722.91	1443.82	722.41	1489.83	745.42	1472.80	736.90	1471.82	**736.41**	A	**376.22**	188.61	359.19	180.10	358.21	179.61	4
16	44.05	1532.87	766.94	1515.84	758.42	1514.86	757.93	1560.86	780.94	1543.84	772.42	1542.85	771.93	A	**305.18**	153.09	288.16	144.58	287.17	144.09	3
17	60.04	1619.90	810.45	1602.87	801.94	1601.89	801.45	1647.90	824.45	1630.87	815.94	1629.88	815.45	S	**234.14**	117.58	217.12	109.06	216.13	108.57	2
18	101.11													K	147.11	74.06	130.09	65.55			1

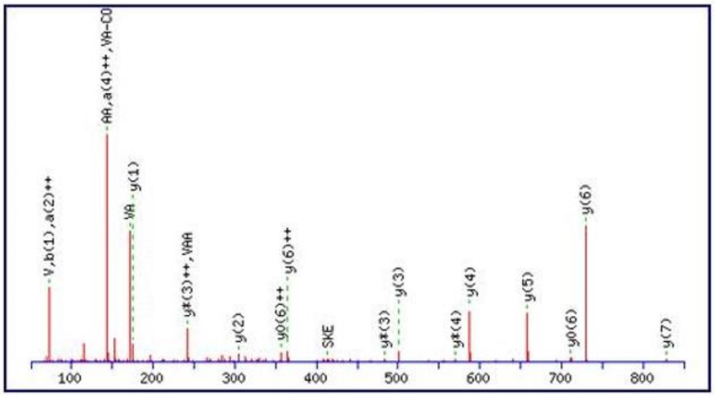

H1K51mea

MS/MS Fragmentation of **AVAASKER**
Found in **P10412**, Histone H1.4

Match to Query 194: 898.486870 from(450.250711,2+)

Monoisotopic mass of neutral peptide Mr(calc): 898.49
Variable modifications:
K6 : Methacryl (K)
Ions Score: 60 **Expect:** 0.00038
Matches (Bold Red): 25/126 fragment ions using 24 most intense peaks

#	Immon.	a	a++	a*	a*++	a0	a0++	b	b++	b*	b*++	b0	b0++	Seq.	y	y++	y*	y*++	y0	y0++	#
1	44.05	44.05	22.53					**72.04**	36.53					A							8
2	**72.08**	**143.12**	**72.06**					**171.11**	86.06					V	**828.46**	414.73	811.43	406.22	810.45	405.73	7
3	44.05	214.15	107.58					**242.15**	121.58					A	**729.39**	**365.20**	712.36	356.68	**711.38**	**356.19**	6
4	44.05	285.19	**143.10**					313.19	157.10					A	**658.35**	329.68	641.33	321.17	640.34	320.67	5
5	60.04	372.22	186.62			354.21	177.61	400.22	200.61			382.21	191.61	S	**587.31**	294.16	**570.29**	285.65	569.30	285.16	4
6	169.13	568.35	284.68	551.32	276.16	550.33	275.67	596.34	298.67	579.31	290.16	578.33	289.67	K	**500.28**	250.64	**483.26**	**242.13**	482.27	241.64	3
7	102.05	697.39	349.20	680.36	340.68	679.38	340.19	725.38	363.20	708.36	354.68	707.37	354.19	E	**304.16**	152.58	287.13	144.07	286.15	143.58	2
8	129.11													R	**175.12**	88.06	158.09	79.55			1

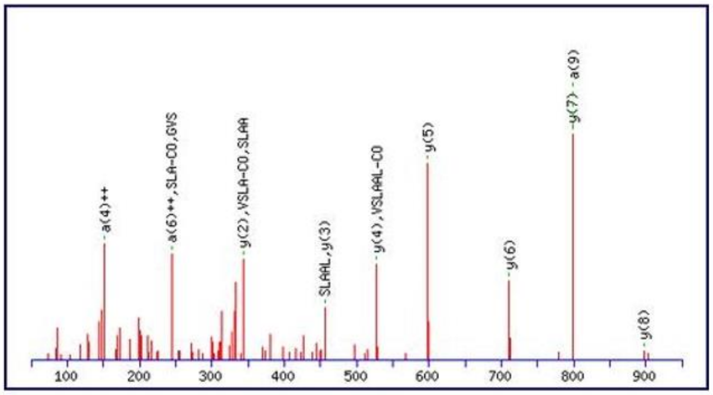

H1K62mea

MS/MS Fragmentation of **SGVSLAALKK**
Found in **P10412**, Histone H1.4

Match to Query 496: 1040.622854 from(521.318703,2+)

Monoisotopic mass of neutral peptide Mr(calc): 1040.62
Variable modifications:
K10 : Methacryl (K)
Ions Score: 78 **Expect:** 7.2e-006
Matches (Bold Red): 17/182 fragment ions using 10 most intense peaks

Should be K9

#	Immon.	a	a^{++}	a^{*}	a^{*++}	a^{0}	a^{0++}	b	b^{++}	b^{*}	b^{*++}	b^{0}	b^{0++}	Seq.	y	y^{++}	y^{*}	y^{*++}	y^{0}	y^{0++}	#
1	60.04	60.04	30.53			42.03	21.52	88.04	44.52			70.03	35.52	S							10
2	30.03	117.07	59.04			99.06	50.03	145.06	73.03			127.05	64.03	G	954.60	477.80	937.57	469.29	936.59	468.80	9
3	72.08	216.13	108.57			198.12	99.57	**244.13**	122.57			226.12	113.56	V	**897.58**	449.29	880.55	440.78	879.57	440.29	8
4	60.04	303.17	**152.09**			285.16	143.08	331.16	166.08			313.15	157.08	S	**798.51**	399.76	781.48	391.24	780.50	390.75	7
5	86.10	416.25	208.63			398.24	199.62	444.25	222.63			426.23	213.62	L	**711.48**	356.24	694.45	347.73			6
6	44.05	487.29	**244.15**			469.28	235.14	515.28	258.14			497.27	249.14	A	**598.39**	299.70	581.37	291.19			5
7	44.05	558.32	279.67			540.31	270.66	586.32	293.66			568.31	284.66	A	**527.36**	264.18	510.33	255.67			4
8	86.10	671.41	336.21			653.40	327.20	699.40	350.21			681.39	341.20	L	**456.32**	228.66	439.29	220.15			3
9	101.11	**799.50**	400.26	782.48	391.74	781.49	391.25	827.50	414.25	810.47	405.74	809.49	405.25	K	**343.23**	172.12	326.21	163.61			2
10	169.13													K	215.14	108.07	198.11	99.56			1

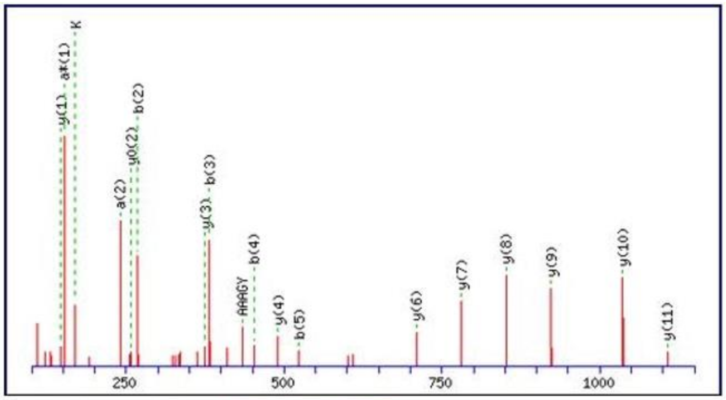

H1K63mea

MS/MS Fragmentation of **KALAAAGYDVEK**
Found in **P10412**, Histone H1.4

Match to Query 944: 1302.681970 from(652.348261,2+)

Monoisotopic mass of neutral peptide Mr(calc): 1302.68
Variable modifications:
K1 : Methacryl (K)
Ions Score: 70 **Expect:** 7.3e-005
Matches (Bold Red): 19/253 fragment ions using 28 most intense peaks

#	Immon.	a	a^{++}	a^{*}	a^{*++}	a^{0}	a^{0++}	b	b^{++}	b^{*}	b^{*++}	b^{0}	b^{0++}	Seq.	y	y^{++}	y^{*}	y^{*++}	y^{0}	y^{0++}	#
1	**169.13**	**169.13**	85.07	**152.11**	76.56			197.13	99.07	180.10	90.55			K							12
2	44.05	**240.17**	120.59	223.14	112.08			**268.17**	134.59	251.14	126.07			A	**1107.57**	554.29	1090.54	545.77	1089.56	545.28	11
3	86.10	353.25	177.13	336.23	168.62			**381.25**	191.13	364.22	182.62			L	**1036.53**	518.77	1019.50	510.26	1018.52	509.76	10
4	44.05	424.29	212.65	407.27	204.14			**452.29**	226.65	435.26	218.13			A	**923.45**	462.23	906.42	453.71	905.44	453.22	9
5	44.05	495.33	248.17	478.30	239.65			**523.32**	262.17	506.30	253.65			A	**852.41**	426.71	835.38	418.20	834.40	417.70	8
6	44.05	566.37	283.69	549.34	275.17			594.36	297.68	577.33	289.17			A	**781.37**	391.19	764.35	382.68	763.36	382.18	7
7	30.03	623.39	312.20	606.36	303.68			651.38	326.19	634.36	317.68			G	**710.34**	355.67	693.31	347.16	692.32	346.67	6
8	136.08	786.45	393.73	769.42	385.22			814.45	407.73	797.42	399.21			Y	653.31	327.16	636.29	318.65	635.30	318.16	5
9	88.04	901.48	451.24	884.45	442.73	883.47	442.24	929.47	465.24	912.45	456.73	911.46	456.23	D	**490.25**	245.63	473.22	237.12	472.24	236.62	4
10	72.08	1000.55	500.78	983.52	492.26	982.54	491.77	1028.54	514.77	1011.51	506.26	1010.53	505.77	V	**375.22**	188.12	358.20	179.60	357.21	179.11	3
11	102.05	1129.59	565.30	1112.56	556.78	1111.58	556.29	1157.58	579.30	1140.56	570.78	1139.57	570.29	E	276.16	138.58	259.13	130.07	**258.14**	129.58	2
12	101.11													K	**147.11**	74.06	130.09	65.55			1

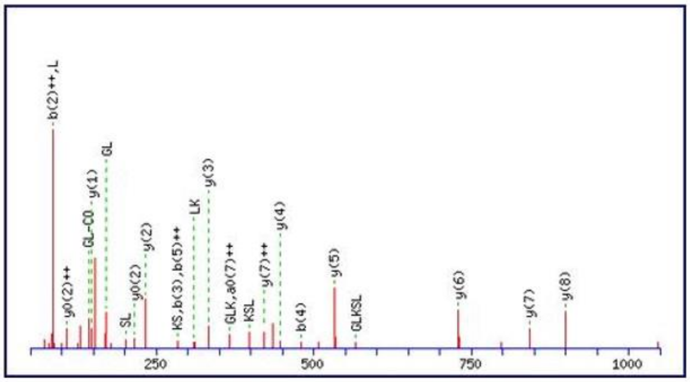

H1K84mea

MS/MS Fragmentation of LGLKSLVSK
Found in P10412, Histone H1.4

Match to Query 432: 1011.631672 from(506.823112,2+)

Monoisotopic mass of neutral peptide Mr(calc): 1011.63
Variable modifications:
K4 : Methacryl (K)
Ions Score: 59 **Expect:** 0.00045
Matches (Bold Red): 32/163 fragment ions using 32 most intense peaks

#	Immon.	a	a^{++}	a^*	a^{*++}	a^0	a^{0++}	b	b^{++}	b^*	b^{*++}	b^0	b^{0++}	Seq.	y	y^{++}	y^*	y^{*++}	y^0	y^{0++}	#
1	**86.10**	**86.10**	43.55					114.09	57.55					L							9
2	30.03	**143.12**	72.06					**171.11**	**86.06**					G	**899.56**	450.28	882.53	441.77	881.55	441.28	8
3	**86.10**	256.20	128.60					**284.20**	142.60					L	**842.53**	**421.77**	825.51	413.26	824.52	412.77	7
4	169.13	452.32	226.67	435.30	218.15			**480.32**	240.66	463.29	232.15			K	**729.45**	365.23	712.42	356.72	711.44	356.22	6
5	60.04	539.36	270.18	522.33	261.67	521.34	261.18	**567.35**	**284.18**	550.32	275.67	549.34	275.17	S	**533.33**	267.17	516.30	258.66	515.32	258.16	5
6	**86.10**	652.44	326.72	635.41	318.21	634.43	317.72	680.43	340.72	663.41	332.21	662.42	331.72	L	**446.30**	223.65	429.27	215.14	428.29	214.65	4
7	72.08	751.51	376.26	734.48	367.74	733.50	**367.25**	779.50	390.25	762.48	381.74	761.49	381.25	V	**333.21**	167.11	316.19	158.60	315.20	158.10	3
8	60.04	838.54	419.77	821.51	411.26	820.53	410.77	866.53	433.77	849.51	425.26	848.52	424.77	S	**234.14**	117.58	217.12	109.06	**216.13**	**108.57**	2
9	101.11													K	**147.11**	74.06	130.09	65.55			1

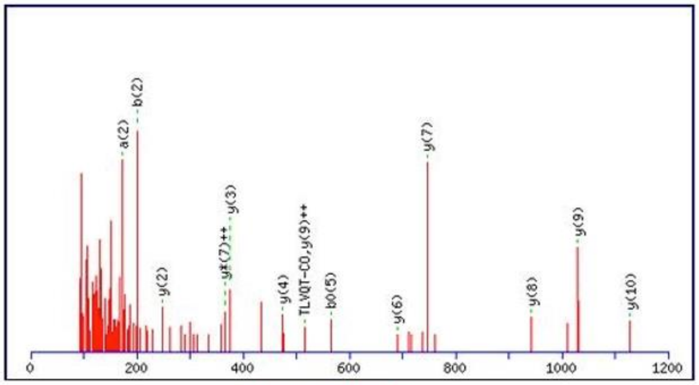

H1K89mea

MS/MS Fragmentation of SLVSKGTLVQTK
Found in P10412, Histone H1.4

Match to Query 990: 1327.769796 from(664.892174,2+)

Monoisotopic mass of neutral peptide Mr(calc): 1327.77
Variable modifications:
K5 : Methacryl (K)
Ions Score: 70 **Expect:** 9.7e-005
Matches (Bold Red): 14/262 fragment ions using 19 most intense peaks

#	Immon.	a	a^{++}	a^*	a^{*++}	a^0	a^{0++}	b	b^{++}	b^*	b^{*++}	b^0	b^{0++}	Seq.	y	y^{++}	y^*	y^{*++}	y^0	y^{0++}	#
1	60.04	60.04	30.53			42.03	21.52	88.04	44.52			70.03	35.52	S							12
2	86.10	**173.13**	87.07			155.12	78.06	**201.12**	101.07			183.11	92.06	L	1241.75	621.38	1224.72	612.86	1223.74	612.37	11
3	72.08	272.20	136.60			254.19	127.60	300.19	150.60			282.18	141.59	V	**1128.66**	564.83	1111.64	556.32	1110.65	555.83	10
4	60.04	359.23	180.12			341.22	171.11	387.22	194.12			369.21	185.11	S	**1029.59**	**515.30**	1012.57	506.79	1011.58	506.30	9
5	169.13	555.35	278.18	538.32	269.67	537.34	269.17	583.34	292.18	566.32	283.66	**565.33**	283.17	K	**942.56**	471.78	925.54	463.27	924.55	462.78	8
6	30.03	612.37	306.69	595.34	298.18	594.36	297.68	640.37	320.69	623.34	312.17	622.36	311.68	G	**746.44**	373.72	729.41	**365.21**	728.43	364.72	7
7	74.06	713.42	357.21	696.39	348.70	695.41	348.21	741.41	371.21	724.39	362.70	723.40	362.21	T	**689.42**	345.21	672.39	336.70	671.41	336.21	6
8	86.10	826.50	413.76	809.48	405.24	808.49	404.75	854.50	427.75	837.47	419.24	836.49	418.75	L	588.37	294.69	571.34	286.18	570.36	285.68	5
9	72.08	925.57	463.29	908.55	454.78	907.56	454.28	953.57	477.29	936.54	468.77	935.56	468.28	V	**475.29**	238.15	458.26	229.63	457.28	229.14	4
10	101.07	1053.63	527.32	1036.60	518.81	1035.62	518.31	1081.63	541.32	1064.60	532.80	1063.61	532.31	Q	**376.22**	188.61	359.19	180.10	358.21	179.61	3
11	74.06	1154.68	577.84	1137.65	569.33	1136.67	568.84	1182.67	591.84	1165.65	583.33	1164.66	582.83	T	**248.16**	124.58	231.13	116.07	230.15	115.58	2
12	101.11													K	147.11	74.06	130.09	65.55			1

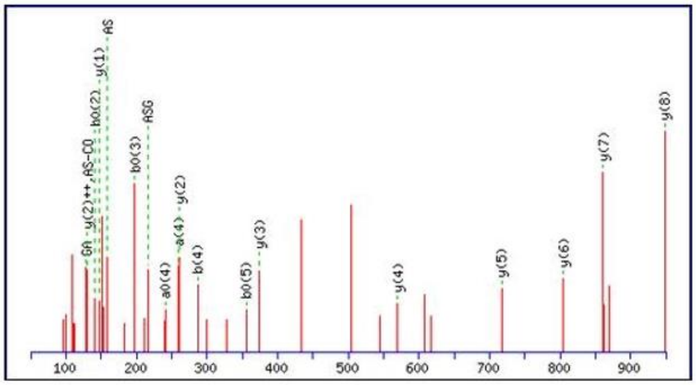

H1K105mea

MS/MS Fragmentation of **GTGASGSFKLNK**
Found in **P10412**, Histone H1.4

Match to Query 802: 1233.635636 from(617.825094,2+)

Monoisotopic mass of neutral peptide Mr(calc): 1233.64
Variable modifications:
K9 : Methacryl (K)
Ions Score: 56 **Expect:** 0.0017
Matches (Bold Red): 26/236 fragment ions using 30 most intense peaks

#	Immon.	a	a^{++}	a^{*}	a^{*++}	a^{0}	a^{0++}	b	b^{++}	b^{*}	b^{*++}	b^{0}	b^{0++}	Seq.	y	y^{++}	y^{*}	y^{*++}	y^{0}	y^{0++}	#
1	30.03	30.03	15.52					58.03	29.52					G							12
2	74.06	**131.08**	66.04			113.07	57.04	**159.08**	80.04			**141.07**	71.04	T	1177.62	589.31	1160.59	580.80	1159.61	580.31	11
3	30.03	188.10	94.56			170.09	85.55	**216.10**	108.55			**198.09**	99.55	G	1076.57	538.79	1059.55	530.28	1058.56	529.79	10
4	44.05	**259.14**	130.07			**241.13**	121.07	**287.13**	144.07			269.12	135.07	A	1019.55	510.28	1002.53	501.77	1001.54	501.27	9
5	60.04	346.17	173.59			328.16	164.58	374.17	187.59			**356.16**	178.58	S	**948.51**	474.76	931.49	466.25	930.50	465.76	8
6	30.03	403.19	202.10			385.18	193.10	431.19	**216.10**			413.18	207.09	G	**861.48**	431.25	844.46	422.73	843.47	422.24	7
7	60.04	490.23	245.62			472.22	236.61	518.22	259.61			500.21	250.61	S	**804.46**	402.73	787.43	394.22	786.45	393.73	6
8	120.08	637.29	319.15			619.28	310.15	665.29	333.15			647.28	324.14	F	**717.43**	359.22	700.40	350.71			5
9	169.13	833.42	417.21	816.39	408.70	815.40	408.21	861.41	431.21	844.38	422.70	843.40	422.20	K	**570.36**	285.68	553.33	277.17			4
10	86.10	946.50	473.75	929.47	465.24	928.49	464.75	974.49	487.75	957.47	479.24	956.48	478.75	L	**374.24**	187.62	357.21	179.11			3
11	87.06	1060.54	530.77	1043.52	522.26	1042.53	521.77	1088.54	544.77	1071.51	536.26	1070.53	535.77	N	**261.16**	**131.08**	244.13	122.57			2
12	101.11													K	**147.11**	74.06	130.09	65.55			1

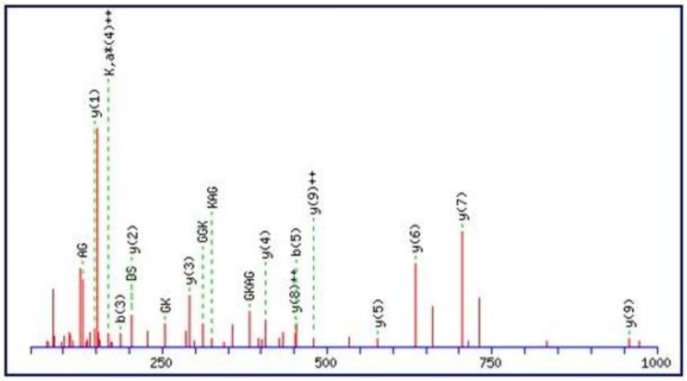

H2AZK4mea

MS/MS Fragmentation of **AGGKAGKDSGK**
Found in **P0C0S5**, Histone H2A.Z

Match to Query 562: 1084.551322 from(543.282937,2+)
[illegible]

Monoisotopic mass of neutral peptide Mr(calc): 1084.55
Variable modifications:
K4 : Methacryl (K)
K7 : Acetyl (K)
Ions Score: 45 **Expect:** 0.011
Matches (Bold Red): 25/208 fragment ions using 38 most intense peaks

#	Immon.	a	a^{++}	a^{*}	a^{*++}	a^{0}	a^{0++}	b	b^{++}	b^{*}	b^{*++}	b^{0}	b^{0++}	Seq.	y	y^{++}	y^{*}	y^{*++}	y^{0}	y^{0++}	#
1	44.05	44.05	22.53					72.04	36.53					A							11
2	30.03	101.07	51.04					**129.07**	65.04					G	1014.52	507.76	997.49	499.25	996.51	498.76	10
3	30.03	158.09	79.55					**186.09**	93.55					G	**957.50**	**479.25**	940.47	470.74	939.49	470.25	9
4	**169.13**	354.21	177.61	337.19	**169.10**			**382.21**	191.61	365.18	183.09			K	900.48	**450.74**	883.45	442.23	882.47	441.74	8
5	44.05	425.25	213.13	408.22	204.62			**453.25**	227.13	436.22	218.61			A	**704.36**	352.68	687.33	344.17	686.35	343.68	7
6	30.03	482.27	241.64	465.25	233.13			510.27	255.64	493.24	247.12			G	**633.32**	317.16	616.29	308.65	615.31	308.16	6
7	143.12	652.38	326.69	635.35	318.18			680.37	340.69	663.35	332.18			K	**576.30**	288.65	559.27	280.14	558.29	279.65	5
8	88.04	767.40	384.21	750.38	375.69	749.39	375.20	795.40	398.20	778.37	389.69	777.39	389.20	D	**406.19**	203.60	389.17	195.09	388.18	194.59	4
9	60.04	854.44	427.72	837.41	419.21	836.43	418.72	882.43	441.72	865.40	433.21	864.42	432.71	S	**291.17**	146.09	274.14	137.57	273.16	137.08	3
10	30.03	911.46	456.23	894.43	447.72	893.45	447.23	939.45	470.23	922.43	461.72	921.44	461.22	G	**204.13**	102.57	187.11	94.06			2
11	101.11													K	**147.11**	74.06	130.09	65.55			1

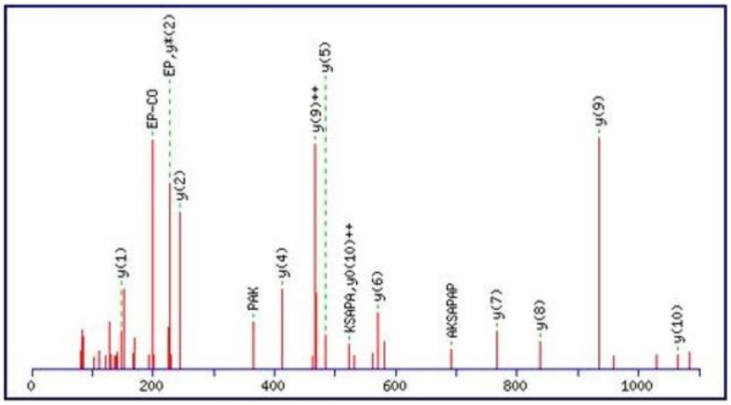

H2BK5mea

MS/MS Fragmentation of PEPAKSAPAPK
Found in P06899, Histone H2B type 1-J

Match to Query 685: 1159.623604 from(580.819078,2+)

Monoisotopic mass of neutral peptide Mr(calc): 1159.62
Variable modifications:
K5 : Methacryl (K)
Ions Score: 65 **Expect:** 0.00027
Matches (Bold Red): 22/225 fragment ions using 27 most intense peaks

#	Immon.	a	a^{++}	a^{*}	a^{*++}	a^{0}	a^{0++}	b	b^{++}	b^{*}	b^{*++}	b^{0}	b^{0++}	Seq.	y	y^{++}	y^{*}	y^{*++}	y^{0}	y^{0++}	#
1	70.07	70.07	35.54					98.06	49.53					P							11
2	102.05	**199.11**	100.06			181.10	91.05	**227.10**	114.05			209.09	105.05	E	**1063.58**	532.29	1046.55	523.78	1045.57	**523.29**	10
3	70.07	296.16	148.58			278.15	139.58	324.16	162.58			306.14	153.58	P	**934.54**	**467.77**	917.51	459.26	916.52	458.77	9
4	44.05	367.20	184.10			349.19	175.10	395.19	198.10			377.18	189.09	A	**837.48**	419.25	820.46	410.73	819.47	410.24	8
5	169.13	563.32	282.16	546.29	273.65	545.31	273.16	591.31	296.16	574.29	287.65	573.30	287.16	K	**766.45**	383.73	749.42	375.21	748.44	374.72	7
6	60.04	650.35	325.68	633.32	317.17	632.34	316.67	678.35	339.68	661.32	331.16	660.34	330.67	S	**570.32**	285.67	553.30	277.15	552.31	276.66	6
7	44.05	721.39	361.20	704.36	352.68	703.38	352.19	749.38	375.20	732.36	366.68	731.37	366.19	A	**483.29**	242.15	466.27	233.64			5
8	70.07	818.44	409.72	801.41	401.21	800.43	400.72	846.44	423.72	829.41	415.21	828.42	414.72	P	**412.26**	206.63	395.23	198.12			4
9	44.05	889.48	445.24	872.45	436.73	871.47	436.24	917.47	459.24	900.45	450.73	899.46	450.23	A	315.20	158.10	298.18	149.59			3
10	70.07	986.53	493.77	969.50	485.26	968.52	484.76	1014.53	507.77	997.50	499.25	996.51	498.76	P	**244.17**	122.59	**227.14**	114.07			2
11	101.11													K	**147.11**	74.06	130.09	65.55			1

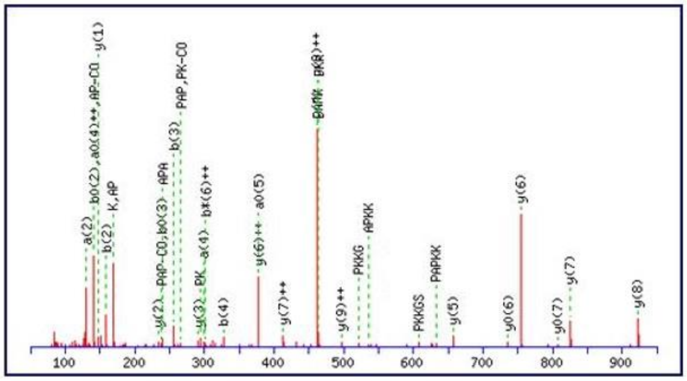

H2BK11mea

MS/MS Fragmentation of SAPAPKKGSK
Found in P06899, Histone H2B type 1-J

Match to Query 555: 1079.596942 from(540.805747,2+)

Monoisotopic mass of neutral peptide Mr(calc): 1079.60
Variable modifications:
K6 : Methacryl (K)
K7 : Acetyl (K)
Ions Score: 44 **Expect:** 0.019
Matches (Bold Red): 42/199 fragment ions using 57 most intense peaks

#	Immon.	a	a^{++}	a^{*}	a^{*++}	a^{0}	a^{0++}	b	b^{++}	b^{*}	b^{*++}	b^{0}	b^{0++}	Seq.	y	y^{++}	y^{*}	y^{*++}	y^{0}	y^{0++}	#
1	60.04	60.04	30.53			42.03	21.52	88.04	44.52			70.03	35.52	S							10
2	44.05	**131.08**	66.04			113.07	57.04	**159.08**	80.04			**141.07**	71.04	A	993.57	**497.29**	976.55	488.78	975.56	488.28	9
3	70.07	228.13	114.57			210.12	105.57	**256.13**	128.57			**238.12**	119.56	P	**922.54**	**461.77**	905.51	453.26	904.52	452.77	8
4	44.05	**299.17**	150.09			281.16	**141.08**	**327.17**	164.09			309.16	155.08	A	**825.48**	**413.25**	808.46	404.73	**807.47**	404.24	7
5	70.07	396.22	198.62			**378.21**	189.61	424.22	212.61			406.21	203.61	P	**754.45**	**377.73**	737.42	369.21	**736.44**	368.72	6
6	**169.13**	592.35	296.68	575.32	288.16	574.33	287.67	620.34	310.67	603.31	**302.16**	602.33	301.67	K	**657.39**	329.20	640.37	320.69	639.38	320.19	5
7	143.12	762.45	381.73	745.42	373.22	744.44	372.72	790.45	395.73	773.42	387.21	772.44	386.72	K	**461.27**	231.14	444.25	222.63	443.26	222.13	4
8	30.03	819.47	410.24	802.45	401.73	801.46	401.23	847.47	424.24	830.44	415.72	829.46	415.23	G	**291.17**	146.09	274.14	137.57	273.16	137.08	3
9	60.04	906.50	453.76	889.48	445.24	888.49	444.75	934.50	467.75	917.47	459.24	916.49	458.75	S	**234.14**	117.58	217.12	109.06	216.13	108.57	2
10	101.11													K	**147.11**	74.06	130.09	65.55			1

H2BK85mea

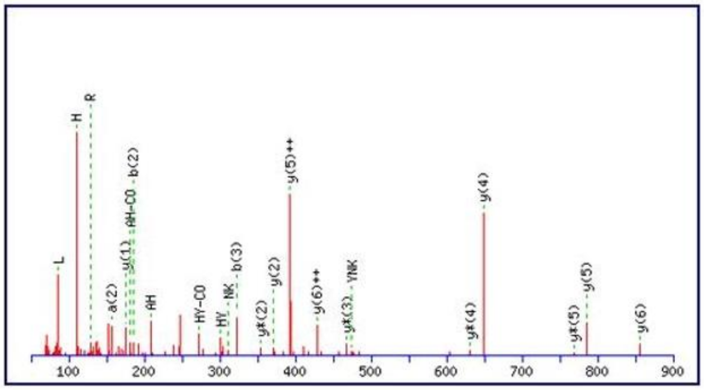

MS/MS Fragmentation of **LAHYNKR**
Found in **P06899**, Histone H2B type 1-J

Match to Query 370: 968.519236 from(485.266894,2+)

Monoisotopic mass of neutral peptide Mr(calc): 968.52
Variable modifications:
K6 : Methacryl (K)
Ions Score: 21 **Expect:** 2.5
Matches (Bold Red): 24/83 fragment ions using 44 most intense peaks

#	Immon.	a	a++	a*	a*++	b	b++	b*	b*++	Seq.	y	y++	y*	y*++	#
1	**86.10**	**86.10**	43.55			114.09	57.55			L					7
2	44.05	**157.13**	79.07			**185.13**	93.07			A	**856.44**	**428.72**	839.42	420.21	6
3	**110.07**	294.19	147.60			**322.19**	161.60			H	**785.41**	**393.21**	**768.38**	384.69	5
4	136.08	457.26	229.13			485.25	243.13			Y	**648.35**	324.68	**631.32**	316.16	4
5	87.06	571.30	286.15	554.27	277.64	599.29	300.15	582.27	291.64	N	485.28	243.15	**468.26**	234.63	3
6	169.13	767.42	384.21	750.39	375.70	795.41	398.21	778.39	389.70	K	**371.24**	186.12	**354.21**	177.61	2
7	**129.11**									R	**175.12**	88.06	158.09	79.55	1

H2BK108mea

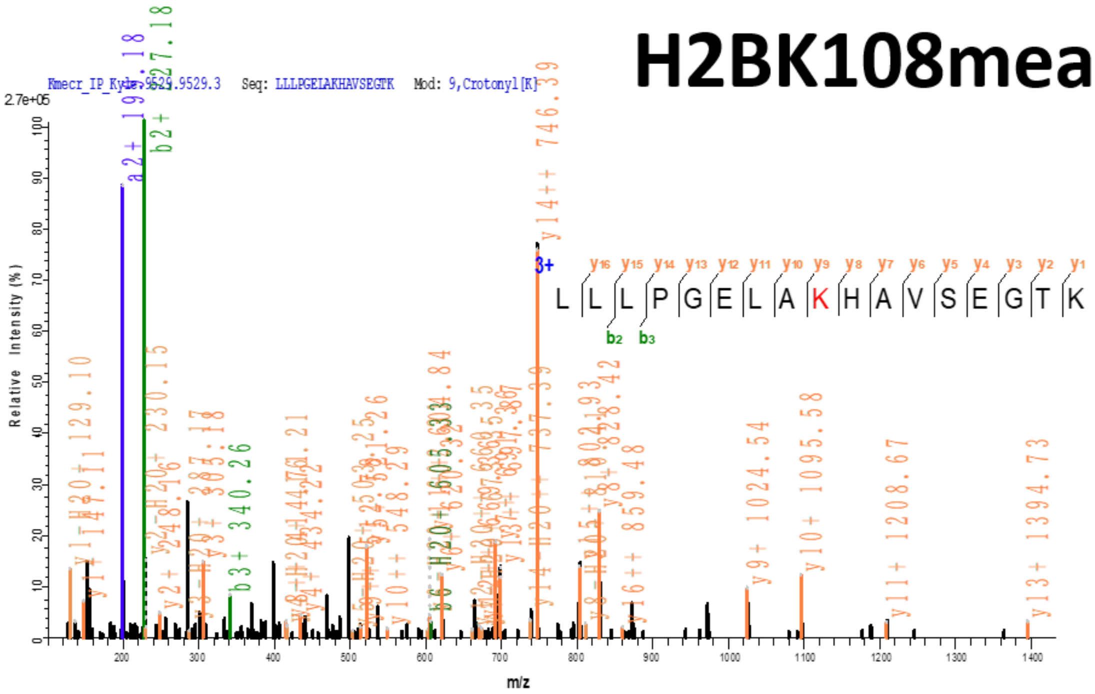

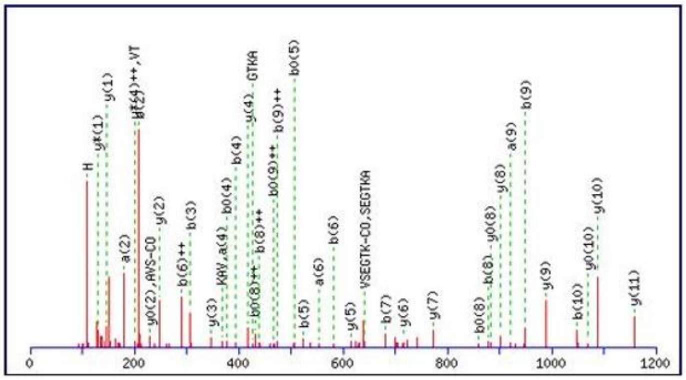

H2BK116mea

MS/MS Fragmentation of HAVSEGTKAVTK
Found in P06899, Histone H2B type 1-J

Match to Query 934: 1294.687784 from(648.351168,2+)

Monoisotopic mass of neutral peptide Mr(calc): 1294.69
Variable modifications:
K8 : Methacryl (K)
Ions Score: 85 **Expect:** 2.1e-006
Matches (Bold Red): 45/238 fragment ions using 64 most intense peaks

#	Immon.	a	a^{++}	a^{*}	a^{*++}	a^{0}	a^{0++}	b	b^{++}	b^{*}	b^{*++}	b^{0}	b^{0++}	Seq.	y	y^{++}	y^{*}	y^{*++}	y^{0}	y^{0++}	#
1	**110.07**	**110.07**	55.54					138.07	69.54					H							12
2	44.05	**181.11**	91.06					**209.10**	105.06					A	**1158.64**	579.82	1141.61	571.31	1140.63	570.82	11
3	72.08	280.18	140.59					**308.17**	154.59					V	**1087.60**	544.30	1070.57	535.79	**1069.59**	535.30	10
4	60.04	**367.21**	184.11			349.20	175.10	**395.20**	198.11			**377.19**	189.10	S	**988.53**	494.77	971.50	486.26	970.52	485.76	9
5	102.05	496.25	248.63			478.24	239.62	**524.25**	262.63			**506.24**	253.62	E	**901.50**	451.25	884.47	442.74	**883.49**	442.25	8
6	30.03	**553.27**	277.14			535.26	268.13	**581.27**	**291.14**			563.26	282.13	G	**772.46**	386.73	755.43	378.22	754.45	377.73	7
7	74.06	654.32	327.66			636.31	318.66	**682.32**	341.66			664.30	332.66	T	**715.43**	358.22	698.41	349.71	697.42	349.22	6
8	169.13	850.44	425.72	833.42	417.21	832.43	416.72	**878.44**	**439.72**	861.41	431.21	**860.43**	**430.72**	K	**614.39**	307.70	597.36	299.18	596.38	298.69	5
9	44.05	**921.48**	461.24	904.45	452.73	903.47	452.24	**949.47**	**475.24**	932.45	466.73	931.46	**466.24**	A	**418.27**	209.64	401.24	**201.12**	400.26	200.63	4
10	72.08	1020.55	510.78	1003.52	502.26	1002.54	501.77	**1048.54**	524.77	1031.52	516.26	1030.53	515.77	V	**347.23**	174.12	330.20	165.60	329.22	165.11	3
11	74.06	1121.59	561.30	1104.57	552.79	1103.58	552.30	1149.59	575.30	1132.56	566.79	1131.58	566.29	T	**248.16**	124.58	231.13	116.07	**230.15**	115.58	2
12	101.11													K	**147.11**	74.06	**130.09**	65.55			1

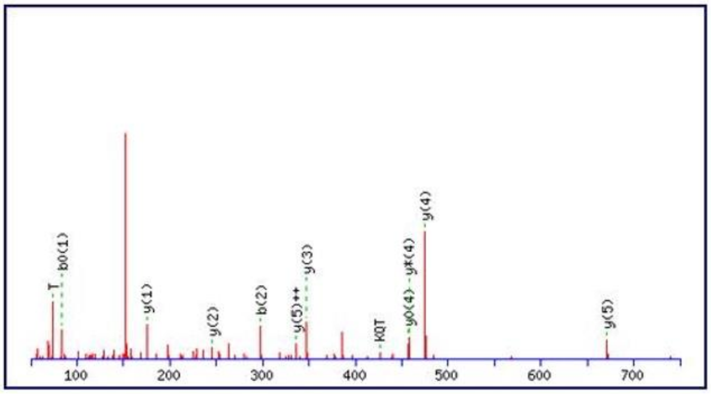

H3K4mea

MS/MS Fragmentation of TKQTAR
Found in Q71DI3, Histone H3.2

Match to Query 17: 771.423906 from(386.719229,2+)

Monoisotopic mass of neutral peptide Mr(calc): 771.42
Variable modifications:
K2 : Methacryl (K)
Ions Score: 37 **Expect:** 0.091
Matches (Bold Red): 15/100 fragment ions using 23 most intense peaks

#	Immon.	a	a^{++}	a^{*}	a^{*++}	a^{0}	a^{0++}	b	b^{++}	b^{*}	b^{*++}	b^{0}	b^{0++}	Seq.	y	y^{++}	y^{*}	y^{*++}	y^{0}	y^{0++}	#
1	**74.06**	**74.06**	37.53			56.05	28.53	102.05	51.53			**84.04**	42.53	T							6
2	169.13	270.18	135.59	253.15	127.08	252.17	126.59	**298.18**	149.59	281.15	141.08	280.17	140.59	K	**671.38**	**336.20**	654.36	327.68	653.37	327.19	5
3	101.07	398.24	199.62	381.21	191.11	380.23	190.62	**426.23**	213.62	409.21	205.11	408.22	204.62	Q	**475.26**	238.13	**458.24**	229.62	**457.25**	229.13	4
4	**74.06**	499.29	250.15	482.26	241.63	481.28	241.14	527.28	264.14	510.26	255.63	509.27	255.14	T	**347.20**	174.11	330.18	165.59	329.19	165.10	3
5	44.05	570.32	285.67	553.30	277.15	552.31	276.66	598.32	299.66	581.29	291.15	580.31	290.66	A	**246.16**	123.58	229.13	115.07			2
6	129.11													R	**175.12**	88.06	158.09	79.55			1

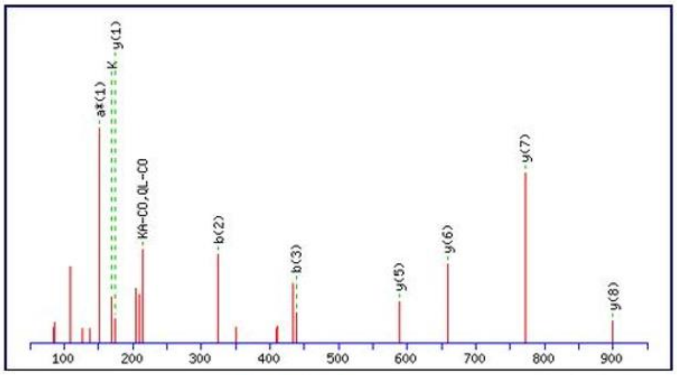

H3K18mea

MS/MS Fragmentation of **KQLATKAAR**
Found in **Q71DI3**, Histone H3.2

Match to Query 582: 1095.639848 from(548.827200,2+)

Monoisotopic mass of neutral peptide Mr(calc): 1095.64
Variable modifications:
K1 : Methacryl (K)
K6 : Acetyl (K)
Ions Score: 28 **Expect:** 0.72
Matches (Bold Red): 12/170 fragment ions using 19 most intense peaks

#	Immon.	a	a^{++}	a^*	a^{*++}	a^0	a^{0++}	b	b^{++}	b^*	b^{*++}	b^0	b^{0++}	Seq.	y	y^{++}	y^*	y^{*++}	y^0	y^{0++}	#
1	**169.13**	**169.13**	85.07	**152.11**	76.56			197.13	99.07	180.10	90.55			K							9
2	101.07	297.19	149.10	280.17	140.59			**325.19**	163.10	308.16	154.58			Q	**900.53**	450.77	883.50	442.25	882.52	441.76	8
3	86.10	410.28	205.64	393.25	197.13			**438.27**	219.64	421.24	211.13			L	**772.47**	386.74	755.44	378.22	754.46	377.73	7
4	44.05	481.31	241.16	464.29	232.65			509.31	255.16	492.28	246.64			A	**659.38**	330.20	642.36	321.68	641.37	321.19	6
5	74.06	582.36	291.68	565.33	283.17	564.35	282.68	610.36	305.68	593.33	297.17	592.35	296.68	T	**588.35**	294.68	571.32	286.16	570.34	285.67	5
6	143.12	752.47	376.74	735.44	368.22	734.46	367.73	780.46	390.73	763.43	382.22	762.45	381.73	K	487.30	244.15	470.27	235.64			4
7	44.05	823.50	412.26	806.48	403.74	805.49	403.25	851.50	426.25	834.47	417.74	833.49	417.25	A	317.19	159.10	300.17	150.59			3
8	44.05	894.54	447.77	877.51	439.26	876.53	438.77	922.54	461.77	905.51	453.26	904.52	452.77	A	246.16	123.58	229.13	115.07			2
9	129.11													R	**175.12**	88.06	158.09	79.55			1

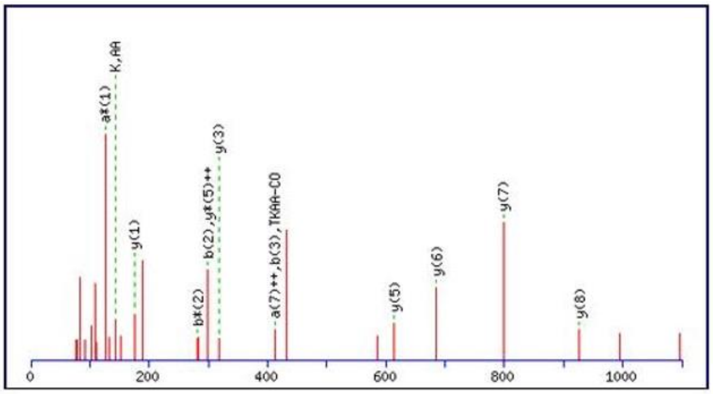

H3K23mea

MS/MS Fragmentation of **KQLATKAAR**
Found in **Q71DI3**, Histone H3.2

Match to Query 580: 1095.639692 from(548.827122,2+)

Monoisotopic mass of neutral peptide Mr(calc): 1095.64
Variable modifications:
K1 : Acetyl (K)
K6 : Methacryl (K)
Ions Score: 41 **Expect:** 0.041
Matches (Bold Red): 17/169 fragment ions using 19 most intense peaks

#	Immon.	a	a^{++}	a^*	a^{*++}	a^0	a^{0++}	b	b^{++}	b^*	b^{*++}	b^0	b^{0++}	Seq.	y	y^{++}	y^*	y^{*++}	y^0	y^{0++}	#
1	**143.12**	**143.12**	72.06	**126.09**	63.55			171.11	86.06	154.09	77.55			K							9
2	101.07	271.18	136.09	254.15	127.58			**299.17**	150.09	**282.14**	141.58			Q	**926.54**	463.77	909.52	455.26	908.53	454.77	8
3	86.10	384.26	192.63	367.23	184.12			**412.26**	206.63	395.23	198.12			L	**798.48**	399.75	781.46	391.23	780.47	390.74	7
4	44.05	455.30	228.15	438.27	219.64			483.29	242.15	466.27	233.64			A	**685.40**	343.20	668.37	334.69	667.39	334.20	6
5	74.06	556.35	278.68	539.32	270.16	538.33	269.67	584.34	292.67	567.31	284.16	566.33	283.67	T	**614.36**	307.68	597.34	**299.17**	596.35	298.68	5
6	169.13	752.47	376.74	735.44	368.22	734.46	367.73	780.46	390.73	763.43	382.22	762.45	381.73	K	513.31	257.16	496.29	248.65			4
7	44.05	823.50	**412.26**	806.48	403.74	805.49	403.25	851.50	426.25	834.47	417.74	833.49	417.25	A	**317.19**	159.10	300.17	150.59			3
8	44.05	894.54	447.77	877.51	439.26	876.53	438.77	922.54	461.77	905.51	453.26	904.52	452.77	A	246.16	123.58	229.13	115.07			2
9	129.11													R	**175.12**	88.06	158.09	79.55			1

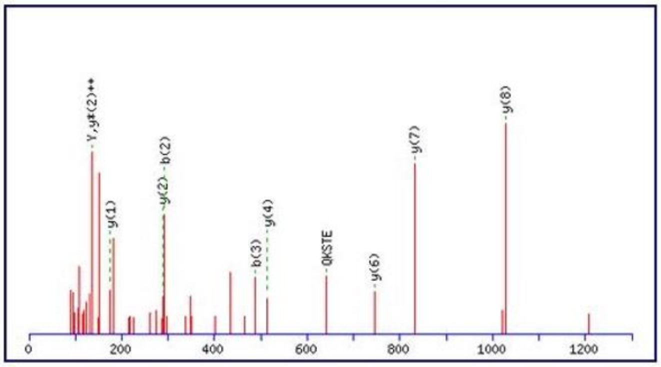

H3K56mea

MS/MS Fragmentation of YQKSTELLIR
Found in Q71DI3, Histone H3.2

Match to Query 962: 1317.730428 from(659.872490,2+)

Monoisotopic mass of neutral peptide Mr(calc): 1317.73
Variable modifications:
K3 : Methacryl (K)
Ions Score: 39 **Expect:** 0.11
Matches (Bold Red): 12/194 fragment ions using 15 most intense peaks

#	Immon.	a	a^{++}	a^{*}	a^{*++}	a^{0}	a^{0++}	b	b^{++}	b^{*}	b^{*++}	b^{0}	b^{0++}	Seq.	y	y^{++}	y^{*}	y^{*++}	y^{0}	y^{0++}	#
1	**136.08**	**136.08**	68.54					164.07	82.54					Y							10
2	101.07	264.13	132.57	247.11	124.06			**292.13**	146.57	275.10	138.05			Q	1155.67	578.34	1138.65	569.83	1137.66	569.33	9
3	169.13	460.26	230.63	443.23	222.12			**488.25**	244.63	471.22	236.12			K	**1027.61**	514.31	1010.59	505.80	1009.60	505.31	8
4	60.04	547.29	274.15	530.26	265.63	529.28	265.14	575.28	288.14	558.26	279.63	557.27	279.14	S	**831.49**	416.25	814.47	407.74	813.48	407.25	7
5	74.06	648.34	324.67	631.31	316.16	630.32	315.67	676.33	338.67	659.30	330.16	658.32	329.66	T	**744.46**	372.73	727.43	364.22	726.45	363.73	6
6	102.05	777.38	389.19	760.35	380.68	759.37	380.19	805.37	403.19	788.35	394.68	787.36	394.18	E	643.41	322.21	626.39	313.70	625.40	313.21	5
7	86.10	890.46	445.73	873.44	437.22	872.45	436.73	918.46	459.73	901.43	451.22	900.45	450.73	L	**514.37**	257.69	497.34	249.18			4
8	86.10	1003.55	502.28	986.52	493.76	985.54	493.27	1031.54	516.27	1014.51	507.76	1013.53	507.27	L	401.29	201.15	384.26	192.63			3
9	86.10	1116.63	558.82	1099.60	550.31	1098.62	549.81	1144.62	572.82	1127.60	564.30	1126.61	563.81	I	**288.20**	144.61	271.18	**136.09**			2
10	129.11													R	**175.12**	88.06	158.09	79.55			1

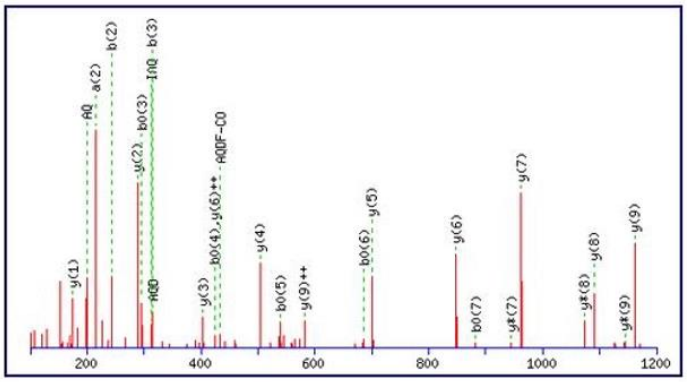

H3K79mea

MS/MS Fragmentation of EIAQDFKTDLR
Found in Q71DI3, Histone H3.2

Match to Query 1092: 1402.709062 from(702.361807,2+)

Monoisotopic mass of neutral peptide Mr(calc): 1402.71
Variable modifications:
K7 : Methacryl (K)
Ions Score: 69 **Expect:** 8.7e-005
Matches (Bold Red): 26/227 fragment ions using 34 most intense peaks

#	Immon.	a	a^{++}	a^{*}	a^{*++}	a^{0}	a^{0++}	b	b^{++}	b^{*}	b^{*++}	b^{0}	b^{0++}	Seq.	y	y^{++}	y^{*}	y^{*++}	y^{0}	y^{0++}	#
1	102.05	102.05	51.53			84.04	42.53	130.05	65.53			112.04	56.52	E							11
2	86.10	**215.14**	108.07			197.13	99.07	**243.13**	122.07			225.12	113.07	I	1274.67	637.84	1257.65	629.33	1256.66	628.84	10
3	44.05	286.18	143.59			268.17	134.59	**314.17**	157.59			**296.16**	148.58	A	**1161.59**	**581.30**	**1144.56**	572.79	1143.58	572.29	9
4	101.07	414.23	207.62	397.21	199.11	396.22	198.62	442.23	221.62	425.20	213.11	**424.22**	212.61	Q	**1090.55**	545.78	**1073.53**	537.27	1072.54	536.77	8
5	88.04	529.26	265.13	512.24	256.62	511.25	256.13	557.26	279.13	540.23	270.62	**539.25**	270.13	D	**962.49**	481.75	**945.47**	473.24	944.48	472.75	7
6	120.08	676.33	338.67	659.30	330.16	658.32	329.66	704.32	352.67	687.30	344.15	**686.31**	343.66	F	**847.47**	**424.24**	830.44	415.72	829.46	415.23	6
7	169.13	872.45	436.73	855.42	428.22	854.44	427.72	900.45	450.73	883.42	442.21	**882.44**	441.72	K	**700.40**	350.70	683.37	342.19	682.39	341.70	5
8	74.06	973.50	487.25	956.47	478.74	955.49	478.25	1001.49	501.25	984.47	492.74	983.48	492.25	T	**504.28**	252.64	487.25	244.13	486.27	243.64	4
9	88.04	1088.53	544.77	1071.50	536.25	1070.52	535.76	1116.52	558.76	1099.49	550.25	1098.51	549.76	D	**403.23**	202.12	386.20	193.61	385.22	193.11	3
10	86.10	1201.61	601.31	1184.58	592.80	1183.60	592.30	1229.60	615.31	1212.58	606.79	1211.59	606.30	L	**288.20**	144.61	271.18	136.09			2
11	129.11													R	**175.12**	88.06	158.09	79.55			1

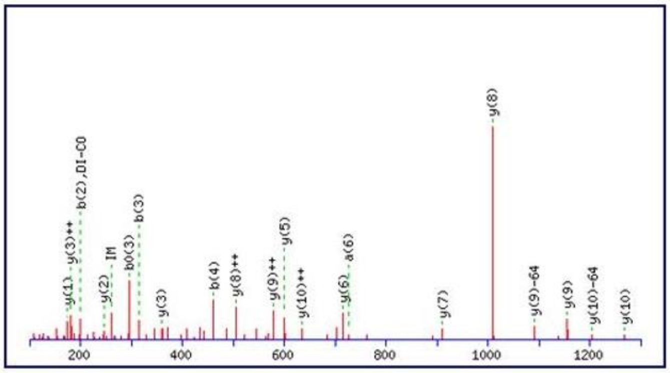

H3K122mea

MS/MS Fragmentation of VTIMPKDIQLAR
Found in Q71DI3, Histone H3.2

Match to Query 1183: 1467.810982 from(734.912767,2+)

Monoisotopic mass of neutral peptide Mr(calc): 1467.81
Variable modifications:
M4 : Oxidation (M), with neutral losses 0.00(shown in table), 64.00
K6 : Methacryl (K)
Ions Score: 68 **Expect:** 0.00013
Matches (Bold Red): 22/365 fragment ions using 31 most intense peaks

#	Immon.	a	a^{++}	a^{*}	a^{*++}	a^{0}	a^{0++}	b	b^{++}	b^{*}	b^{*++}	b^{0}	b^{0++}	Seq.	y	y^{++}	y^{*}	y^{*++}	y^{0}	y^{0++}	#
1	72.08	72.08	36.54					100.08	50.54					V							12
2	74.06	173.13	87.07			155.12	78.06	**201.12**	101.07			183.11	92.06	T	1369.75	685.38	1352.72	676.87	1351.74	676.37	11
3	86.10	286.21	143.61			268.20	134.60	**314.21**	157.61			**296.20**	148.60	I	**1268.70**	**634.86**	1251.68	626.34	1250.69	625.85	10
4	120.05	433.25	217.13			415.24	208.12	**461.24**	231.13			443.23	222.12	M	**1155.62**	**578.31**	1138.59	569.80	1137.61	569.31	9
5	70.07	530.30	265.65			512.29	256.65	558.30	279.65			540.29	270.65	P	**1008.58**	**504.80**	991.56	496.28	990.57	495.79	8
6	169.13	**726.42**	363.71	709.40	355.20	708.41	354.71	754.42	377.71	737.39	369.20	736.41	368.71	K	**911.53**	456.27	894.50	447.76	893.52	447.26	7
7	88.04	841.45	421.23	824.42	412.71	823.44	412.22	869.44	435.23	852.42	426.71	851.43	426.22	D	**715.41**	358.21	698.38	349.70	697.40	349.20	6
8	86.10	954.53	477.77	937.51	469.26	936.52	468.76	982.53	491.77	965.50	483.25	964.52	482.76	I	**600.38**	300.70	583.36	292.18			5
9	101.07	1082.59	541.80	1065.56	533.29	1064.58	532.79	1110.59	555.80	1093.56	547.28	1092.58	546.79	Q	487.30	244.15	470.27	235.64			4
10	86.10	1195.68	598.34	1178.65	589.83	1177.66	589.34	1223.67	612.34	1206.64	603.83	1205.66	603.33	L	**359.24**	**180.12**	342.21	171.61			3
11	44.05	1266.71	633.86	1249.69	625.35	1248.70	624.85	1294.71	647.86	1277.68	639.34	1276.70	638.85	A	**246.16**	123.58	229.13	115.07			2
12	129.11													R	**175.12**	88.06	158.09	79.55			1

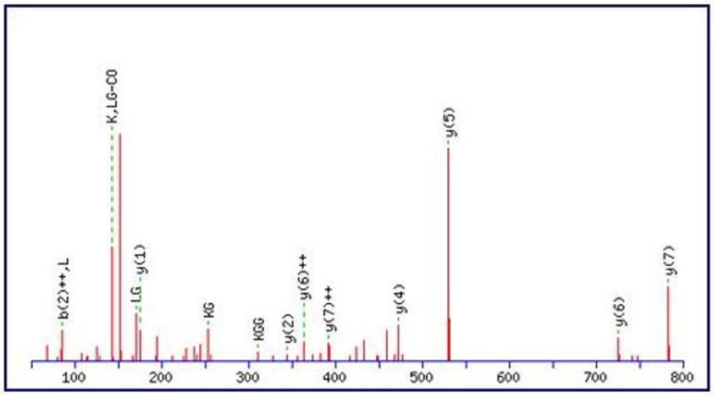

H4K12mea

MS/MS Fragmentation of GLGKGGAKR
Found in P62805, Histone H4

Match to Query 323: 952.545324 from(477.279938,2+)

Monoisotopic mass of neutral peptide Mr(calc): 952.55
Variable modifications:
K4 : Methacryl (K)
K8 : Acetyl (K)
Ions Score: 43 **Expect:** 0.016
Matches (Bold Red): 19/134 fragment ions using 20 most intense peaks

#	Immon.	a	a^{++}	a^{*}	a^{*++}	b	b^{++}	b^{*}	b^{*++}	Seq.	y	y^{++}	y^{*}	y^{*++}	#
1	30.03	30.03	15.52			58.03	29.52			G					9
2	**86.10**	**143.12**	72.06			**171.11**	**86.06**			L	896.53	448.77	879.50	440.26	8
3	30.03	200.14	100.57			228.13	114.57			G	**783.45**	**392.23**	766.42	383.71	7
4	169.13	396.26	198.63	379.23	190.12	424.26	212.63	407.23	204.12	K	**726.43**	**363.72**	709.40	355.20	6
5	30.03	453.28	227.14	436.26	218.63	481.28	241.14	464.25	232.63	G	**530.30**	265.66	513.28	257.14	5
6	30.03	510.30	255.66	493.28	247.14	538.30	269.65	521.27	261.14	G	**473.28**	237.15	456.26	228.63	4
7	44.05	581.34	291.17	564.31	282.66	609.34	305.17	592.31	296.66	A	416.26	208.63	399.24	200.12	3
8	**143.12**	751.45	376.23	734.42	367.71	779.44	390.22	762.41	381.71	K	**345.22**	173.12	328.20	164.60	2
9	129.11									R	**175.12**	88.06	158.09	79.55	1

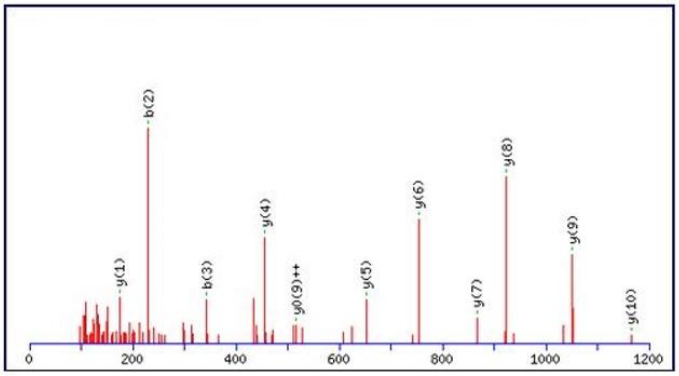

H4K31mea

MS/MS Fragmentation of **DNIQGITKPAIR**
Found in **P62805**, Histone H4

Match to Query 1078: 1392.771922 from(697.393237,2+)

Monoisotopic mass of neutral peptide Mr(calc): 1392.77
Variable modifications:
K8 : Methacryl (K)
Ions Score: 86 **Expect:** 1.8e-006
Matches (Bold Red): 11/265 fragment ions using 14 most intense peaks

#	Immon.	a	a^{++}	a^{*}	a^{*++}	a^{0}	a^{0++}	b	b^{++}	b^{*}	b^{*++}	b^{0}	b^{0++}	Seq.	y	y^{++}	y^{*}	y^{*++}	y^{0}	y^{0++}	#
1	88.04	88.04	44.52			70.03	35.52	116.03	58.52			98.02	49.52	D							12
2	87.06	202.08	101.54	185.06	93.03	184.07	92.54	**230.08**	115.54	213.05	107.03	212.07	106.54	N	1278.75	639.88	1261.73	631.37	1260.74	630.87	11
3	86.10	315.17	158.09	298.14	149.57	297.16	149.08	**343.16**	172.08	326.13	163.57	325.15	163.08	I	**1164.71**	582.86	1147.68	574.35	1146.70	573.85	10
4	101.07	443.22	222.12	426.20	213.60	425.21	213.11	471.22	236.11	454.19	227.60	453.21	227.11	Q	**1051.63**	526.32	1034.60	517.80	1033.62	**517.31**	9
5	30.03	500.25	250.63	483.22	242.11	482.24	241.62	528.24	264.62	511.21	256.11	510.23	255.62	G	**923.57**	462.29	906.54	453.77	905.56	453.28	8
6	86.10	613.33	307.17	596.30	298.66	595.32	298.16	641.33	321.17	624.30	312.65	623.31	312.16	I	**866.55**	433.78	849.52	425.26	848.54	424.77	7
7	74.06	714.38	357.69	697.35	349.18	696.37	348.69	742.37	371.69	725.35	363.18	724.36	362.68	T	**753.46**	377.23	736.44	368.72	735.45	368.23	6
8	169.13	910.50	455.75	893.47	447.24	892.49	446.75	938.49	469.75	921.47	461.24	920.48	460.75	K	**652.41**	326.71	635.39	318.20			5
9	70.07	1007.55	504.28	990.53	495.77	989.54	495.27	1035.55	518.28	1018.52	509.76	1017.54	509.27	P	**456.29**	228.65	439.27	220.14			4
10	44.05	1078.59	539.80	1061.56	531.28	1060.58	530.79	1106.58	553.80	1089.56	545.28	1088.57	544.79	A	359.24	180.12	342.21	171.61			3
11	86.10	1191.67	596.34	1174.65	587.83	1173.66	587.33	1219.67	610.34	1202.64	601.82	1201.66	601.33	I	288.20	144.61	271.18	136.09			2
12	129.11													R	**175.12**	88.06	158.09	79.55			1

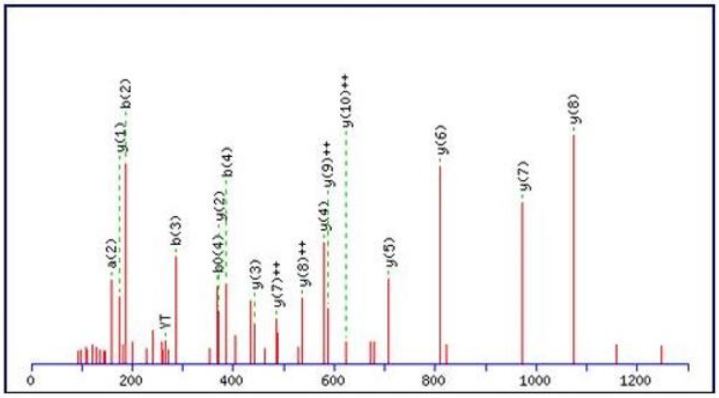

H4K77mea

MS/MS Fragmentation of **DAVTYTEHAKR**
Found in **P62805**, Histone H4

Match to Query 1027: 1357.662022 from(679.838287,2+)

Monoisotopic mass of neutral peptide Mr(calc): 1357.66
Variable modifications:
K10 : Methacryl (K)
Ions Score: 58 **Expect:** 0.00056
Matches (Bold Red): 19/202 fragment ions using 29 most intense peaks

#	Immon.	a	a^{++}	a^{*}	a^{*++}	a^{0}	a^{0++}	b	b^{++}	b^{*}	b^{*++}	b^{0}	b^{0++}	Seq.	y	y^{++}	y^{*}	y^{*++}	y^{0}	y^{0++}	#
1	88.04	88.04	44.52			70.03	35.52	116.03	58.52			98.02	49.52	D							11
2	44.05	**159.08**	80.04			141.07	71.04	**187.07**	94.04			169.06	85.03	A	1243.64	**622.33**	1226.62	613.81	1225.63	613.32	10
3	72.08	258.14	129.58			240.13	120.57	**286.14**	143.57			268.13	134.57	V	1172.61	**586.81**	1155.58	578.29	1154.60	577.80	9
4	74.06	359.19	180.10			341.18	171.09	**387.19**	194.10			**369.18**	185.09	T	**1073.54**	**537.27**	1056.51	528.76	1055.53	528.27	8
5	136.08	522.26	261.63			504.25	252.63	550.25	275.63			532.24	266.62	Y	**972.49**	**486.75**	955.46	478.24	954.48	477.74	7
6	74.06	623.30	312.16			605.29	303.15	651.30	326.15			633.29	317.15	T	**809.43**	405.22	792.40	396.70	791.42	396.21	6
7	102.05	752.35	376.68			734.34	367.67	780.34	390.67			762.33	381.67	E	**708.38**	354.69	691.35	346.18	690.37	345.69	5
8	110.07	889.41	445.21			871.39	436.20	917.40	459.20			899.39	450.20	H	**579.34**	290.17	562.31	281.66			4
9	44.05	960.44	480.72			942.43	471.72	988.44	494.72			970.43	485.72	A	**442.28**	221.64	425.25	213.13			3
10	169.13	1156.56	578.79	1139.54	570.27	1138.55	569.78	1184.56	592.78	1167.53	584.27	1166.55	583.78	K	**371.24**	186.12	354.21	177.61			2
11	129.11													R	**175.12**	88.06	158.09	79.55			1

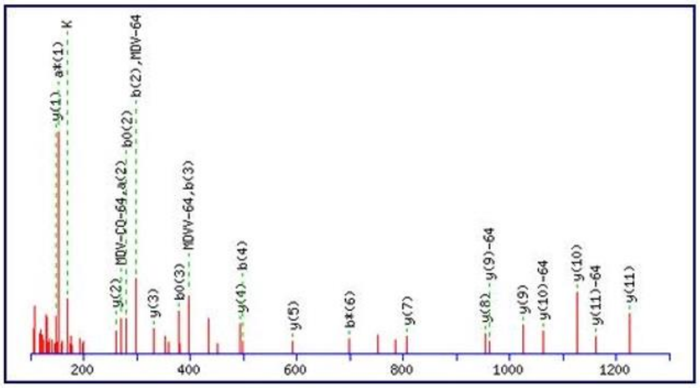

H4K79mea

MS/MS Fragmentation of KTVTAMDVVYALK
Found in P62805, Histone H4

Match to Query 1237: 1521.810238 from(761.912395,2+)

Monoisotopic mass of neutral peptide Mr(calc): 1521.81
Variable modifications:
K1 : Methacryl (K)
M6 : Oxidation (M), with neutral losses 0.00(shown in table), 64.00
Ions Score: 75 **Expect:** 2.9e-005
Matches (Bold Red): 26/446 fragment ions using 34 most intense peaks

#	Immon.	a	a^{++}	a^{*}	a^{*++}	a^{0}	a^{0++}	b	b^{++}	b^{*}	b^{*++}	b^{0}	b^{0++}	Seq.	y	y^{++}	y^{*}	y^{*++}	y^{0}	y^{0++}	#
1	**169.13**	**169.13**	85.07	**152.11**	76.56			197.13	99.07	180.10	90.55			K							13
2	74.06	**270.18**	135.59	253.15	127.08	252.17	126.59	**298.18**	149.59	281.15	141.08	**280.17**	140.59	T	1326.70	663.85	1309.67	655.34	1308.69	654.85	12
3	72.08	369.25	185.13	352.22	176.62	351.24	176.12	**397.24**	199.13	380.22	190.61	**379.23**	190.12	V	**1225.65**	613.33	1208.62	604.82	1207.64	604.32	11
4	74.06	470.30	235.65	453.27	227.14	452.29	226.65	**498.29**	249.65	481.27	241.14	480.28	240.64	T	**1126.58**	563.79	1109.55	555.28	1108.57	554.79	10
5	44.05	541.33	271.17	524.31	262.66	523.32	262.17	569.33	285.17	552.30	276.65	551.32	276.16	A	**1025.53**	513.27	1008.51	504.76	1007.52	504.27	9
6	120.05	688.37	344.69	671.34	336.18	670.36	335.68	716.36	358.69	**699.34**	350.17	698.35	349.68	M	**954.50**	477.75	937.47	469.24	936.49	468.75	8
7	88.04	803.40	402.20	786.37	393.69	785.39	393.20	831.39	416.20	814.37	407.69	813.38	407.19	D	**807.46**	404.23	790.43	395.72	789.45	395.23	7
8	72.08	902.47	451.74	885.44	443.22	884.45	442.73	930.46	465.73	913.43	457.22	912.45	456.73	V	692.43	346.72	675.41	338.21			6
9	72.08	1001.53	501.27	984.51	492.76	983.52	492.27	1029.53	515.27	1012.50	506.75	1011.52	506.26	V	**593.37**	297.19	576.34	288.67			5
10	136.08	1164.60	582.80	1147.57	574.29	1146.59	573.80	1192.59	596.80	1175.57	588.29	1174.58	587.79	Y	**494.30**	247.65	477.27	239.14			4
11	44.05	1235.63	618.32	1218.61	609.81	1217.62	609.32	1263.63	632.32	1246.60	623.80	1245.62	623.31	A	**331.23**	166.12	314.21	157.61			3
12	86.10	1348.72	674.86	1331.69	666.35	1330.71	665.86	1376.71	688.86	1359.69	680.35	1358.70	679.85	L	**260.20**	130.60	243.17	122.09			2
13	101.11													K	**147.11**	74.06	130.09	65.55			1

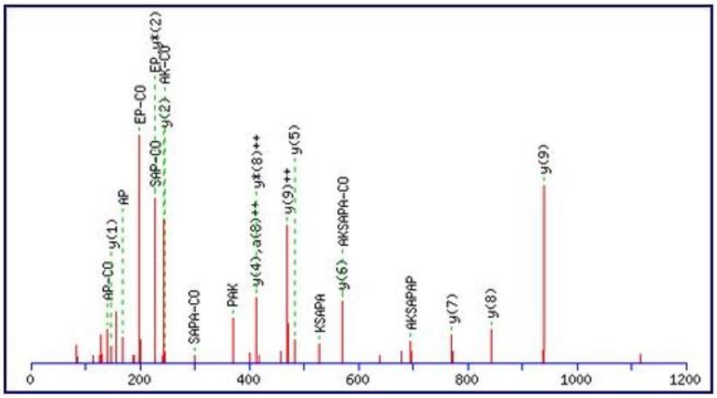

H2BK5mea (D5)

MS/MS Fragmentation of PEPAKSAPAPK
Found in P57053, Histone H2B type F-S

Match to Query 1344: 1164.655338 from(583.334945,2+)

Monoisotopic mass of neutral peptide Mr(calc): 1164.66
Variable modifications:
K5 : Methacryl (K_D5)
Ions Score: 43 **Expect:** 0.038
Matches (Bold Red): 34/225 fragment ions using 35 most intense peaks

#	Immon.	a	a^{++}	a^{*}	a^{*++}	a^{0}	a^{0++}	b	b^{++}	b^{*}	b^{*++}	b^{0}	b^{0++}	Seq.	y	y^{++}	y^{*}	y^{*++}	y^{0}	y^{0++}	#
1	70.07	70.07	35.54					98.06	49.53					P							11
2	102.05	**199.11**	100.06			181.10	91.05	**227.10**	114.05			209.09	105.05	E	1068.61	534.81	1051.58	526.30	1050.60	525.80	10
3	70.07	296.16	148.58			278.15	139.58	324.16	162.58			306.14	153.58	P	**939.57**	**470.29**	922.54	461.77	921.56	461.28	9
4	44.05	367.20	184.10			349.19	175.10	395.19	198.10			377.18	189.09	A	**842.51**	421.76	825.49	**413.25**	824.50	412.76	8
5	174.16	568.35	284.68	551.32	276.17	550.34	275.67	596.34	298.68	579.32	290.16	578.33	289.67	K	**771.48**	386.24	754.45	377.73	753.47	377.24	7
6	60.04	655.38	328.19	638.36	319.68	637.37	319.19	683.38	342.19	666.35	333.68	665.37	333.19	S	**570.32**	285.67	553.30	277.15	552.31	276.66	6
7	44.05	726.42	363.71	709.39	355.20	708.41	354.71	754.41	377.71	737.39	369.20	736.40	368.71	A	**483.29**	242.15	466.27	233.64			5
8	70.07	823.47	**412.24**	806.45	403.73	805.46	403.23	851.47	426.24	834.44	417.72	833.46	417.23	P	**412.26**	206.63	395.23	198.12			4
9	44.05	894.51	447.76	877.48	439.24	876.50	438.75	922.50	461.76	905.48	453.24	904.49	452.75	A	315.20	158.10	298.18	149.59			3
10	70.07	991.56	496.28	974.54	487.77	973.55	487.28	1019.56	510.28	1002.53	501.77	1001.55	501.28	P	**244.17**	122.59	**227.14**	114.07			2
11	101.11													K	**147.11**	74.06	130.09	65.55			1

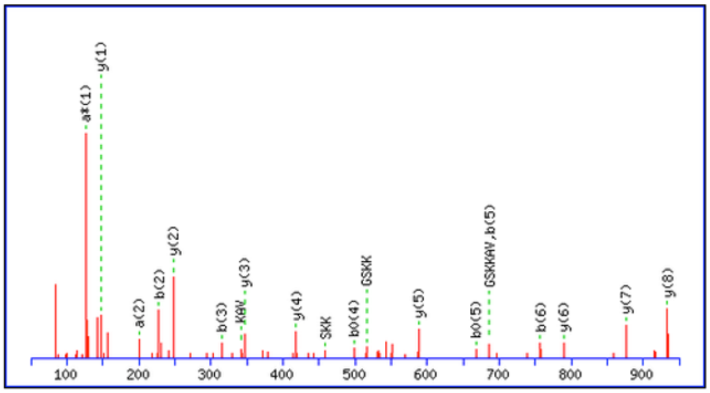

H2BK15mea (D5)

MS/MS Fragmentation of KGSKKAVTK
Found in P06899, Histone H2B type 1-J OS=Homo sapiens GN=HIST1H2BJ PE=1 SV=3

Match to Query 1123: 1102.675192 from(552.344872,2+)

Monoisotopic mass of neutral peptide Mr(calc): 1102.68
Variable modifications:
K1 : Acetyl (K)
K4 : Methacryl (K_D5)
K5 : Acetyl (K)
Ions Score: 64 **Expect:** 9.3e-006
Matches (Bold Red): 21/181 fragment ions using 29 most intense peaks

#	Immon.	a	a^{++}	a^{*}	a^{*++}	a^{0}	a^{0++}	b	b^{++}	b^{*}	b^{*++}	b^{0}	b^{0++}	Seq.	y	y^{++}	y^{*}	y^{*++}	y^{0}	y^{0++}	#
1	143.12	143.12	72.06	**126.09**	63.55			171.11	86.06	154.09	77.55			K							9
2	30.03	**200.14**	100.57	183.11	92.06			**228.13**	114.57	211.11	106.06			G	**933.58**	467.29	916.55	458.78	915.57	458.29	8
3	60.04	287.17	144.09	270.14	135.58	269.16	135.08	**315.17**	158.09	298.14	149.57	297.16	149.08	S	**876.56**	438.78	859.53	430.27	858.55	429.78	7
4	174.16	488.32	244.67	471.30	236.15	470.31	235.66	**516.32**	258.66	499.29	250.15	**498.31**	249.66	K	**789.52**	395.27	772.50	386.75	771.51	386.26	6
5	143.12	658.43	329.72	641.40	321.21	640.42	320.71	**686.42**	343.72	669.40	335.20	**668.41**	334.71	K	**588.37**	294.69	571.34	286.18	570.36	285.68	5
6	44.05	729.47	365.24	712.44	356.72	711.46	356.23	**757.46**	379.23	740.43	370.72	739.45	370.23	A	**418.27**	209.64	401.24	201.12	400.26	200.63	4
7	72.08	828.53	414.77	811.51	406.26	810.52	405.77	856.53	428.77	839.50	420.26	838.52	419.76	V	**347.23**	174.12	330.20	165.60	329.22	165.11	3
8	74.06	929.58	465.29	912.56	456.78	911.57	456.29	957.58	479.29	940.55	470.78	939.57	470.29	T	**248.16**	124.58	231.13	116.07	230.15	115.58	2
9	101.11													K	**147.11**	74.06	130.09	65.55			1

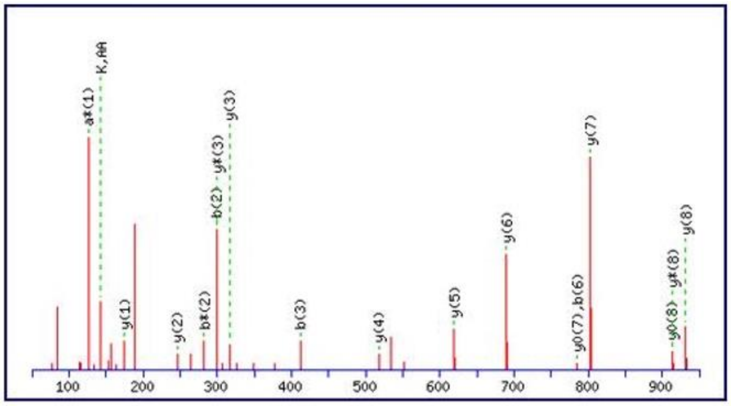

H3K23mea (D5)

MS/MS Fragmentation of KQLATKAAR
Found in Q71DI3, Histone H3.2

Match to Query 1114: 1100.671748 from(551.343150,2+)

Monoisotopic mass of neutral peptide Mr(calc): 1100.67
Variable modifications:
K1 : Acetyl (K)
K6 : Methacryl (K_D5)
Ions Score: 62 **Expect:** 0.00047
Matches (Bold Red): 20/169 fragment ions using 31 most intense peaks

#	Immon.	a	a^{++}	a^{*}	a^{*++}	a^{0}	a^{0++}	b	b^{++}	b^{*}	b^{*++}	b^{0}	b^{0++}	Seq.	y	y^{++}	y^{*}	y^{*++}	y^{0}	y^{0++}	#
1	**143.12**	**143.12**	72.06	**126.09**	63.55			171.11	86.06	154.09	77.55			K							9
2	101.07	271.18	136.09	254.15	127.58			**299.17**	150.09	**282.14**	141.58			Q	**931.57**	466.29	**914.55**	457.78	**913.56**	457.28	8
3	86.10	384.26	192.63	367.23	184.12			**412.26**	206.63	395.23	198.12			L	**803.51**	402.26	786.49	393.75	**785.50**	393.26	7
4	44.05	455.30	228.15	438.27	219.64			483.29	242.15	466.27	233.64			A	**690.43**	345.72	673.40	337.21	672.42	336.71	6
5	74.06	556.35	278.68	539.32	270.16	538.33	269.67	584.34	292.67	567.31	284.16	566.33	283.67	T	**619.39**	310.20	602.37	301.69	601.38	301.20	5
6	174.16	757.50	379.25	740.47	370.74	739.49	370.25	**785.49**	393.25	768.47	384.74	767.48	384.24	K	**518.35**	259.68	501.32	251.16			4
7	44.05	828.53	414.77	811.51	406.26	810.52	405.77	856.53	428.77	839.50	420.26	838.52	419.76	A	**317.19**	159.10	**300.17**	150.59			3
8	44.05	899.57	450.29	882.55	441.78	881.56	441.28	927.57	464.29	910.54	455.77	909.56	455.28	A	**246.16**	123.58	229.13	115.07			2
9	129.11													R	**175.12**	88.06	158.09	79.55			1

Supplementary Fig. S7: Kmea peptide spectra matches.

Annotated MS/MS spectra of Kmea sites identified through IP-HPLC-MS/MS using a pan anti-Kmea antibody for enrichment of tryptically digested HeLa histone extracts. Fragment ions were annotated using MASCOT algorithm with the exception of H2BK108mea, which was identified and annotated using software developed in-house. D5-isotopically labeled peptides due to d7-methacrylate cell treatment are indicated by the annotation (D5) in the peptide name. Not all ions are annotated for all spectra.

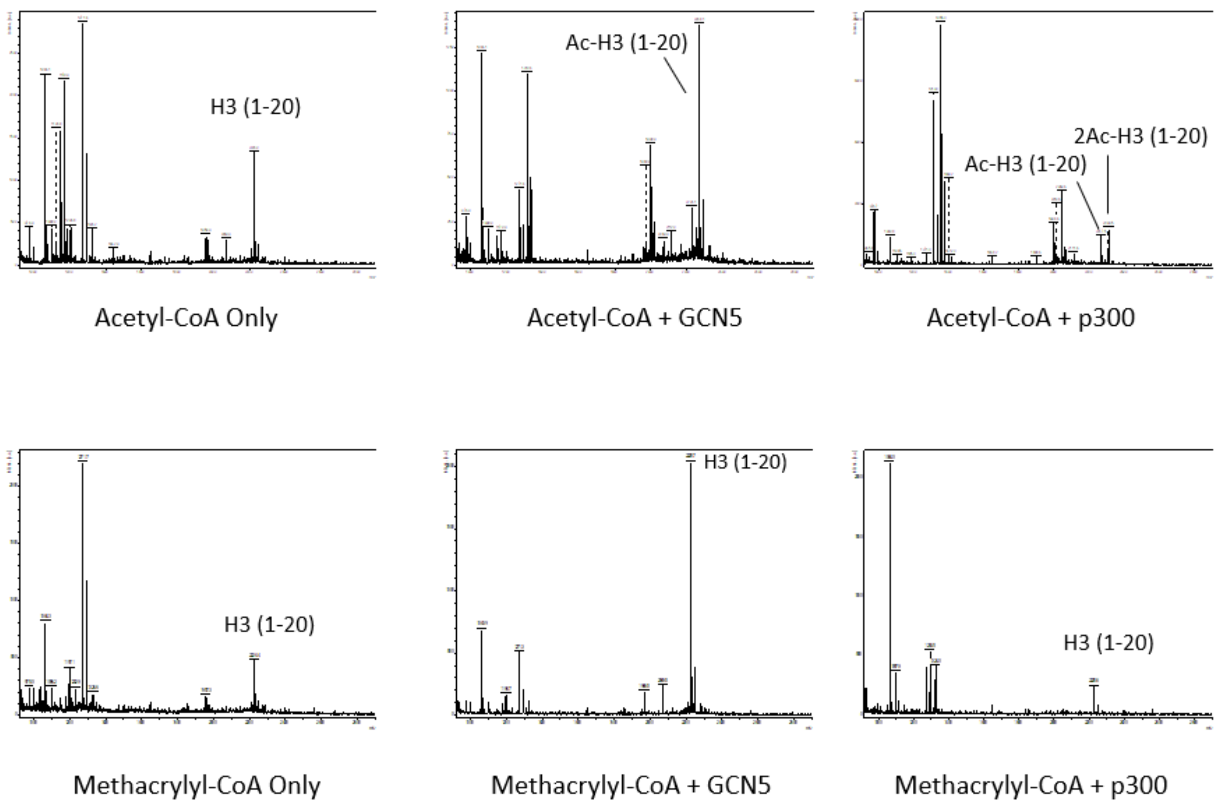

Supplementary Fig. S8: *In vitro* screen of GCN5 and p300 for Kmea writer activity. The synthetic peptide consisting of the first 20 amino acid residues of the H3 histone, H3 (1-20) was incubated with the indicated acyl-CoA and recombinant enzyme *in vitro*. The samples were subjected to MALDI-TOF analysis to detect modified peptide forms.

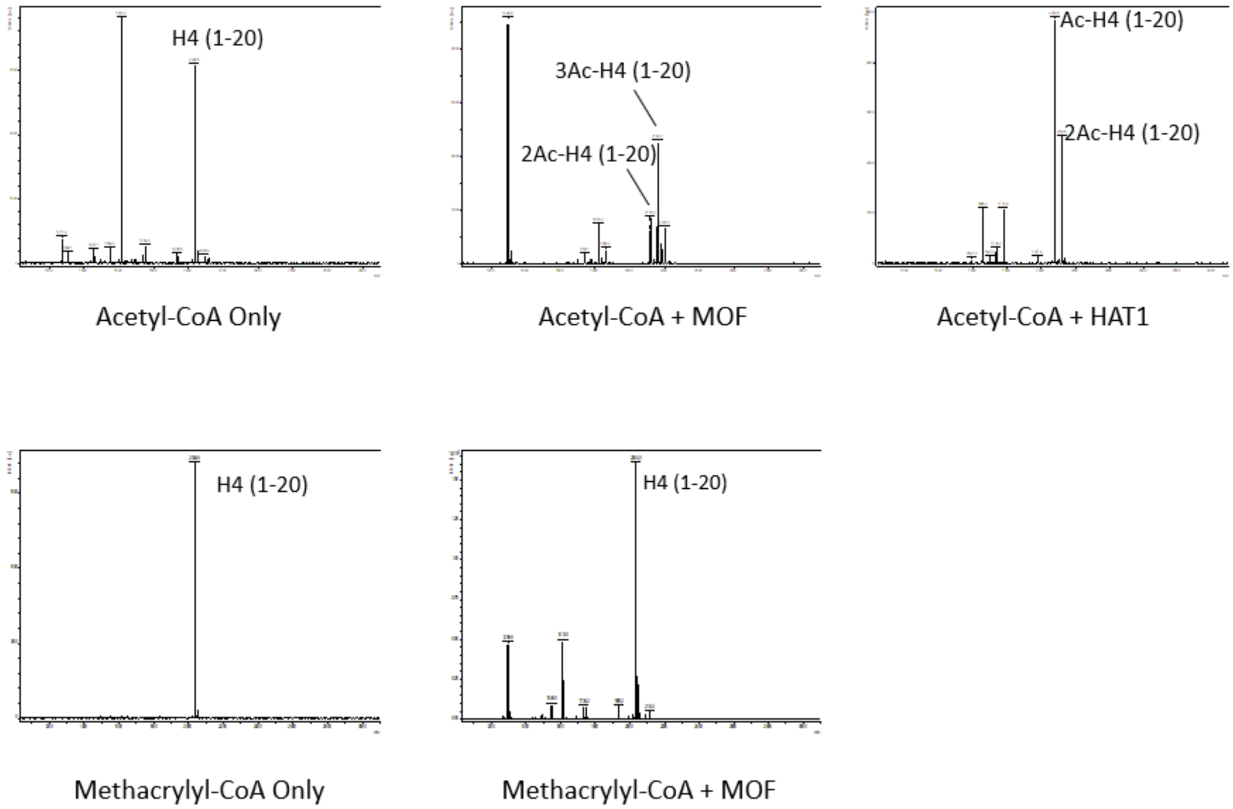

Supplementary Fig. S9: *In vitro* screen of MOF and HAT1 for Kmea writer activity. The synthetic peptides consisting of the first 20 amino acid residues of the H4 histone, H4 (1-20) was incubated with the indicated acyl-CoA and recombinant enzyme *in vitro*. The samples were subjected to MALDI-TOF analysis to detect modified peptide forms.
